# The amplitude of fNIRS hemodynamic response in the visual cortex unmasks autistic traits in typically developing children

**DOI:** 10.1038/s41398-022-01820-5

**Published:** 2022-02-08

**Authors:** Raffaele Mazziotti, Elena Scaffei, Eugenia Conti, Viviana Marchi, Riccardo Rizzi, Giovanni Cioni, Roberta Battini, Laura Baroncelli

**Affiliations:** 1 Department of Developmental Neuroscience, IRCCS Stella Maris Foundation, I-56128 Pisa, Italy; 2 Department of Neuroscience, Psychology, Drug Research and Child Health NEUROFARBA, University of Florence, I-50135 Florence, Italy; 3 Department of Clinical and Experimental Medicine, University of Pisa, Pisa, Italy; 4 Institute of Neuroscience, National Research Council (CNR), I-56124 Pisa, Italy

**Keywords:** Diagnostic markers, Autism spectrum disorders

## Abstract

Autistic traits represent a continuum dimension across the population, with autism spectrum disorder (ASD) being the extreme end of the distribution. Accumulating evidence shows that neuroanatomical and neurofunctional profiles described in relatives of ASD individuals reflect an intermediate neurobiological pattern between the clinical population and healthy controls. This suggests that quantitative measures detecting autistic traits in the general population represent potential candidates for the development of biomarkers identifying early pathophysiological processes associated with ASD. Functional near-infrared spectroscopy (fNIRS) has been extensively employed to investigate neural development and function. In contrast, the potential of fNIRS to define reliable biomarkers of brain activity has been barely explored. Features of non-invasiveness, portability, ease of administration, and low-operating costs make fNIRS a suitable instrument to assess brain function for differential diagnosis, follow-up, analysis of treatment outcomes, and personalized medicine in several neurological conditions. Here, we introduce a novel standardized procedure with high entertaining value to measure hemodynamic responses (HDR) in the occipital cortex of adult subjects and children. We found that the variability of evoked HDR correlates with the autistic traits of children, assessed by the Autism-Spectrum Quotient. Interestingly, HDR amplitude was especially linked to social and communication features, representing the core symptoms of ASD. These findings establish a quick and easy strategy for measuring visually-evoked cortical activity with fNIRS that optimize the compliance of young subjects, setting the background for testing the diagnostic value of fNIRS visual measurements in the ASD clinical population.

## Introduction

Autism spectrum disorder (ASD) is a heterogeneous developmental condition that involves persistent challenges in social interactions, restricted/repetitive behaviors, and the lack of behavioral and cognitive flexibility [1]. Since the pioneering work by Lorna Wing [2], increasing epidemiological evidence indicates that autistic traits are continuously distributed across the general population [3, 4]. This is due to the complex genetic and epigenetic inheritance pattern of ASD, where multiple candidate loci contribute to the pathogenesis of the disease [5, 6]. Milder autistic traits have been termed the extended or broader autism phenotype (BAP), with BAP features being particularly prevalent in first- and second-degree relatives of individuals with ASD [6–9]. Much research is currently focused on siblings of children diagnosed with ASD, namely high-risk infants (HR), because around 20% of them receive a diagnosis of ASD within the third year of life [10] and a further 20–30% develop neurodevelopmental conditions [11]. Moreover, a high rate of BAP symptoms has been documented in this population [12]. The prospective study of HR children allows to detect behavioral risk signs or biomarkers of neurodevelopmental disorders at a very early age, before the full-blown clinical expression, while the investigation of BAP features in the general population might be helpful to dissect clinical subtypes and set-up personalized intervention strategies according to developmental stages.

Over the last decade, the biological dimension of ASD has been largely explored, thanks to the growing availability of advanced tools to explore brain correlates of neurological disorders, including high-density EEG, magnetoencephalography, positron emission tomography, magnetic resonance imaging (MRI), and functional near-infrared spectroscopy (fNIRS) [8, 13, 14]. A number of studies reported defective neuroanatomical and neurofunctional features in individuals with ASD, suggesting that a dysfunction of specific brain areas might underlie the core symptoms of ASD [15–19]. Interestingly, relatives of autistic probands, even when not behaviorally impaired, display neurostructural and neurofunctional patterns significantly different from healthy controls and correlated to BAP features [8]. Since ASD and broader autistic manifestations share common genetic variants and neurobiological susceptibility factor [20], the general population emerges as a suitable testing bed for the development of quantitative measures detecting hallmarks of autism.

FNIRS is an optical imaging technique that allows quantifying oxygen consumption in different regions of the cerebral cortex, providing an indirect measure of neuronal activity [21, 22]. This blood-oxygen-level-dependent (BOLD) signal is similar to that detected with functional MRI (fMRI) [23]. However, fNIRS is more tolerant to motion artifacts than fMRI, and the development of robust methods for motion detection and correction allowed to avoid sedation in children [24, 25]. Furthermore, fNIRS has the advantage of being totally non-invasive, low-cost, portable, noiseless, and endowed with high experimental flexibility and no setting constraints. This methodological strength provides the fNIRS with a high ecological value for investigating neural circuit maturation either in typically developing children or clinically relevant populations [24, 26].

Although the use of fNIRS in autism research is still an emerging area, a number of studies aiming to decipher the neuronal mechanisms and circuits underlying ASD evaluated different aspects of brain function and organization, including resting-state and task-evoked responses [27, 28]. Coherence analyses of resting-state hemodynamic activity showed weaker local and interhemispheric functional connectivity in different cortical regions [29–34]. Moreover, individuals on the autism spectrum present patterns of atypical activity, including reduced hemodynamic responses within specific brain regions, bilateral differences in neuronal activation, and the lack of cortical specialization, in tasks ranging from sensory perception [35] to executive functions [36], social perception [37–40], joint attention [41–43], imitation [44, 45], facial and emotional processing [46–49], speech perception and language [50–53]. The majority of studies targeting evoked brain activity were focused on the prefrontal and the temporal cortex [28], where symptom severity seems to be inversely correlated with the degree of cortical activation [45, 46].

Growing evidence suggests that fNIRS might be a candidate biomarker for several neuropsychiatric disorders, including ASD [32, 54–58]. In particular, functional network efficiency [32], weighted separability of NIRS signals [59], multi-layer neural networks and sample entropy of spontaneous hemodynamic fluctuations [57, 60] have been proposed as auxiliary diagnosis indexes for ASD. However, all these approaches require complex algorithms to extract high-level features from the fNIRS raw data, while fitness, applicability, and translational value of biomarkers greatly depend on their ease of use. In this framework, the analysis of visual phenotype has become an important model to evaluate cortical processing in different neurodevelopmental conditions [61–66]. Indeed, electrophysiological measurement of visually evoked responses has been introduced as a quantitative method to assess brain function in Rett syndrome [61, 67], and hemodynamic responses (HDR) emerged as a potential longitudinal biomarker for CDKL5 Deficiency Disorder and Creatine Transporter Deficiency in murine models [65, 66].

Since clinical studies suggested a dysregulation of sensory processing and functional connectivity in the visual cortex of ASD subjects [68–70], and atypical visual processing has been implicated in the neurobiology of autism [71, 72], we hypothesized that fNIRS visual measures might represent a tool to quantitively assess inter-individual differences in autistic traits. The stimulation protocol used in most fNIRS and electrophysiological studies quantitively assessing visual responses in both typical and clinical populations consisted of reversing black and white checkerboard patterns spaced by baseline intervals with a grey isoluminant screen [67, 71–75]. The same paradigm has been also employed to measure visually evoked potentials in autistic individuals [71, 72]. The entertaining value of the checkerboard/gray alternation, however, is quite low, possibly reducing the experimental compliance of children with intellectual and neurodevelopmental disorders.

Thus, this work had three specific aims: (1) to set up a novel standardized procedure to assess HDR in the occipital cortex; (2) to test the feasibility and reliability of fNIRS measurements in typical adults and children using this innovative stimulation protocol; (3) to investigate the correlation between HDR and broad autism dimensions, evaluated with the Autism-Spectrum Quotient (AQ [76, 77]), in the general population.

## Results

### An animated cartoon-based stimulus is able to evoke visual responses in the adult cortex

**Fig. 1:**
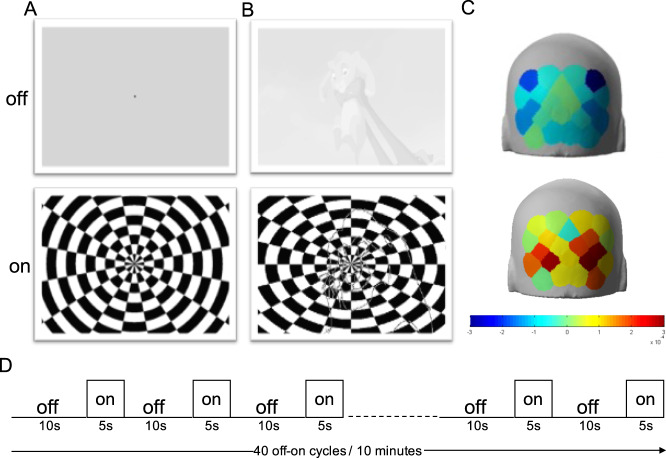
Visual stimulation and experimental paradigm. **A** Representative frame of baseline grey screen (upper row, stimulus ‘off’) and reversing checkerboard (lower row, stimulus ‘on’) for RS condition. The small black square indicates the fixation point. **B** Representative frame of low-contrast (20%) grey-scale baseline animated cartoon (upper row, stimulus ‘off’) and blended checkerboard-cartoon (lower row, stimulus ‘on’) for Cartoon-Fixed (CF) and Cartoon Chosen (CC) conditions. **C** Representative HDR in the occipital cortex during the stimulus ‘off’ (upper row) and stimulus ‘on’ activation phase (lower row) according to the output of nirsLAB software. The Look Up table is reported under the images. **D** Experimental protocol showing that the cycles of visual stimulation were structured in blocks of 40 trials (20 trials with the reversing checkerboard and 20 trials with the ‘mock’ stimulus) for a total duration of 10 min.

**Fig. 2:**
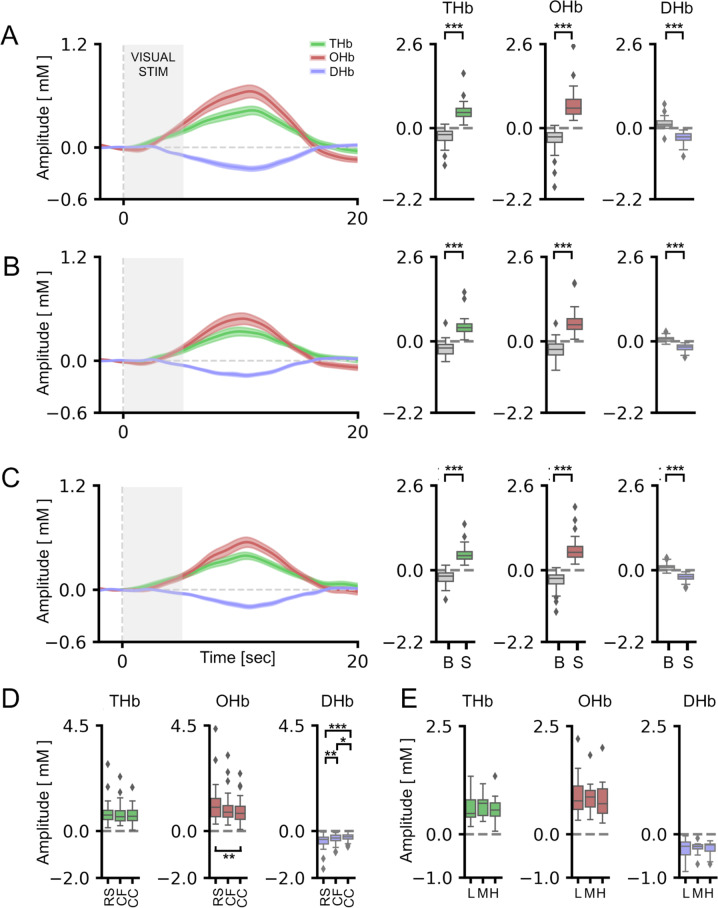
HDR was reliably detected in adults using both RS and blended RS-animated cartoons. For all panels, values in the *y*-axis are multiplied for 10^4. **A** On the left, the average time course for THb (green line), OHb (red line), and DHb (blue line) in response to the Radial Simulus (RS) are shown. The three plots on the right depict the average peak response to the stimulus (S) vs. the blank (B) across all the adult subjects. The stimulus-driven signal was significantly different from the blank for all the conditions (t-test, *p* < 0.001 for all comparisons). **B** Same plots as above for the Cartoon Fixed (CF) condition. On the left, the average time course of the evoked HDR is depicted. On the right, the graphs showed that the HDR amplitude was significantly higher in response to S with respect to B for THb, OHb, and DHb (t-test, *p* < 0.001 for all comparisons). **C** Cartoon Chosen (CC) condition. Also in this case the S elicited significantly higher responses for THb, OHb, and DHb with respect to the B (t-test, *p* < 0.001 for all comparisons). **D** Comparison among different visual stimulations shows no differences in evoked amplitudes for THb, whereas a significant difference was detected between RS and CC for OHb (One-way RM ANOVA, *p* < 0.01, post hoc BH-FDR, RS vs. CC *p* < 0.01) and a more complex pattern of differences emerged for DHb (One-way RM ANOVA, *p* < 0.001, post hoc BH-FDR, RS vs. CF *p* < 0.01, RS vs. CC *p* < 0.001, CF vs CC *p* < 0.05). **E** No differences of evoked responses were detected with different contrast levels of the baseline movie (L: low, M: medium, H: high). For statistical metrics and details, refer to Table S1. Data are shown as average ± s.e.m. * *p* < 0.05; ** *p* < 0.01; *** *p* < 0.001.

We measured the cortical HDR function [78] elicited by a reversing checkerboard pattern in the adult population. In agreement with the previous literature [69, 79], we obtained a significant activation of the occipital cortex in response to different conditions of visual stimulation (Fig. 1 and Fig. S1). Grand averages across adult participants (see Table 1 for demographics) of Total Hb (THb), OxyHb (OHb) and DeoxyHb (DHb) concentration changes are plotted in Fig. 2. Using a classic mean-luminance gray screen as baseline, statistical analysis revealed a significant main effect of the checkerboard stimulus (S) with respect to the blank presentation for all HDR metrics (Radial Stimulus condition, RS, Fig. 2A; see Table S1 for statistical details).

**Table 1:** Demographic characteristics of adult subjects.

ID	Age	Gender	Head	Cap size	AQ	AQ_S	AQ_C	AQ_A	AQ_D	AQ_I	CF	CC
A1	29	F	57	56	11	0	1	7	1	2	The Lion King Walt Disney Pictures,1994	The Sword in the Stone Walt Disney Productions,1963
A2	36	F	56	56	17	0	0	6	9	2	The Powerpuff Girls special episode “Twas the Fight Before Christmas”, Cartoon Network Studios, 2003	The Aristocats Walt Disney Productions,1970
A3	35	M	57	56	17	3	3	5	0	6	Kung Fu Panda DreamWorks Animation, 2008	Aladdin Walt Disney Pictures,1992
A4	28	F	57	56	3	0	0	1	1	1	Peppa Pig “Hide-and-seek”, “Fly the kite”, “Polly parrot” episodes, Entertainment One, 2004	The Simpsons S29E02 “Springfield Splendor”, 20th Television, 2017
A5	25	F	57	56	19	4	1	7	6	1	The Lion King Walt Disney Pictures,1994	101 Dalmatians Walt Disney Productions,1961
A6	35	F	57	56	15	4	2	4	5	0	The Powerpuff Girls special episode “Twas the Fight Before Christmas”, Cartoon Network Studios, 2003	The Simpsons S29E02 “Springfield Splendor”, 20th Television, 2017
A7	29	F	57	56	11	0	2	5	3	1	Peppa Pig “Hide-and-seek”, “Fly the kite”, “Polly parrot” episodes, Entertainment One, 2004	101 Dalmatians Walt Disney Productions,1961
A8	30	F	56	56	13	0	2	4	5	2	Kung Fu Panda DreamWorks Animation, 2008	Wall-E Disney-Pixar, 2008
A9	29	F	59	56	7	1	1	3	2	0	The Lion King Walt Disney Pictures,1994	The Sword in the Stone Walt Disney Productions,1963
A10	29	M	58	56	12	1	1	5	4	1	The Powerpuff Girls special episode “Twas the Fight Before Christmas”, Cartoon Network Studios, 2003	Aladdin Walt Disney Pictures,1992
A11	27	M	56.5	56	17	2	3	5	4	3	Peppa Pig “Hide-and-seek”, “Fly the kite”, “Polly parrot” episodes, Entertainment One, 2004	101 Dalmatians Walt Disney Productions,1961
A12	30	F	56	56	9	1	2	2	3	1	Kung Fu Panda DreamWorks Animation, 2008	Inside out Disney-Pixar, 2015
A13	29	M	57	56	14	1	3	6	2	2	The Lion King Walt Disney Pictures,1994	Wall-E Disney-Pixar, 2008
A14	36	M	58.5	56	19	2	2	5	7	3	The Powerpuff Girls special episode “Twas the Fight Before Christmas”, Cartoon Network Studios, 2003	101 Dalmatians Walt Disney Productions,1961
A15	36	M	57	56	16	4	1	4	5	2	Peppa Pig “Hide-and-seek”, “Fly the kite”, “Polly parrot” episodes, Entertainment One, 2004	Aladdin Walt Disney Pictures,1992
A16	29	F	54	56	22	1	3	9	7	2	Kung Fu Panda DreamWorks Animation, 2008	The Rescuers Walt Disney Productions,1977
A17	28	F	58.5	56	7	1	0	2	1	3	The Lion King Walt Disney Pictures,1994	101 Dalmatians Walt Disney Productions,1961
A18	29	F	55	56	12	0	1	5	5	1	The Powerpuff Girls special episode “Twas the Fight Before Christmas”, Cartoon Network Studios, 2003	Peter Pan Walt Disney Productions,1953
A19	34	M	60	56	19	6	2	5	5	1	Peppa Pig “Hide-and-seek”, “Fly the kite”, “Polly parrot” episodes, Entertainment One, 2004	SpongeBob S12E01 “FarmerBob”, Nickelodeon Animation Studio, 2018
A20	26	M	58	56	10	3	0	5	2	0	Kung Fu Panda DreamWorks Animation, 2008	Inside out Disney-Pixar, 2015
A21	36	F	56	56	15	1	2	2	7	3	The Lion King Walt Disney Pictures,1994	Inside out Disney-Pixar, 2015
A22	30	M	60.5	56	19	1	2	6	4	6	The Lion King Walt Disney Pictures,1994	The Sword in the Stone Walt Disney Productions,1963
A23	26	F	56	56	12	0	1	5	6	0	The Powerpuff Girls special episode “Twas the Fight Before Christmas”, Cartoon Network Studios, 2003	Wall-E Disney-Pixar, 2008
A24	32	M	60.5	56	7	0	1	5	0	1	Kung Fu Panda DreamWorks Animation, 2008	Aladdin Walt Disney Pictures,1992
A25	34	F	56	56	10	1	1	4	2	2	The Lion King Walt Disney Pictures1994	Beauty and the Beast Walt Disney Pictures,1994
A26	29	F	57	56	32	6	5	8	9	4	The Powerpuff Girls special episode “Twas the Fight Before Christmas”, Cartoon Network Studios, 2003	The Aristocats Walt Disney Productions,1970
A27	31	M	59	56	32	10	6	6	2	8	Kung Fu Panda DreamWorks Animation, 2008	The Simpsons S29E02 “Springfield Splendor”, 20th Television, 2017
A28	39	F	57	56	21	2	4	4	8	3	Peppa Pig “Hide-and-seek”, “Fly the kite”, “Polly parrot” episodes, Entertainment One, 2004	Lady Oscar, “A Funeral Bell Tolls in the Twilight” episode, Discotek Media, 1980
A29	29	M	57	56	6	0	0	2	2	2	The Lion King Walt Disney Pictures,1994	The Simpsons S29E02 “Springfield Splendor”, 20th Television, 2017
A30	29	M	57	56	17	3	2	5	6	1	The Powerpuff Girls special episode “Twas the Fight Before Christmas”, Cartoon Network Studios, 2003	The Sword in the Stone Walt Disney Productions,1963
A31	36	M	59.5	56	12	0	0	6	4	2	Peppa Pig “Hide-and-seek”, “Fly the kite”, “Polly parrot” episodes, Entertainment One, 2004	The Sword in the Stone Walt Disney Productions,1963
A32	27	M	59	56	12	1	2	5	2	2	The Powerpuff Girls special episode “Twas the Fight Before Christmas”, Cartoon Network Studios, 2003	The Aristocats Walt Disney Productions,1970
A33	40	M	57	56	13	2	3	1	6	1	Peppa Pig “Hide-and-seek”, “Fly the kite”, “Polly parrot” episodes, Entertainment One, 2004	The Simpsons S29E02 “Springfield Splendor”, 20th Television, 2017
A34	31	F	55	56	15	1	1	4	4	5	Kung Fu Panda DreamWorks Animation, 2008	The Sword in the Stone Walt Disney Productions,1963
A35	27	M	56.5	56	19	6	2	4	7	0	The Lion King Walt Disney Pictures,1994	Frozen Walt Disney Pictures, 2013
A36	30	M	58	56	14	5	1	3	4	1	The Powerpuff Girls special episode “Twas the Fight Before Christmas”, Cartoon Network Studios, 2003	Wall-E Disney-Pixar, 2008
A37	25	M	58	56	29	6	8	6	7	2	Peppa Pig “Hide-and-seek”, “Fly the kite”, “Polly parrot” episodes, Entertainment One, 2004	Wall-E Disney-Pixar, 2008
A38	31	M	57.5	56	10	0	1	5	3	1	Kung Fu Panda DreamWorks Animation, 2008	Inside out Disney-Pixar, 2015
A39	38	M	57.5	56	25	2	4	6	8	5	The Lion King Walt Disney Pictures,1994	The Sword in the Stone Walt Disney Productions,1963
A40	33	F	57.5	56	13	0	0	3	10	0	The Powerpuff Girls special episode “Twas the Fight Before Christmas”, Cartoon Network Studios, 2003	Beauty and the Beast Walt Disney Pictures,1994

Age (years), gender, head circumference (head, cm), cap size (cm), total AQ score (AQ), AQ subscale scores (AQ_S, AQ_C, AQ_A, AQ_D, AQ_I) and the movies used for visual stimulation (CF and CC according to the experimental protocol) are listed for each participant. For movies, production company, release date, and episode title are indicated as well.

To increase the entertaining quality of our experimental paradigm, we devised an innovative visual stimulation protocol blending the checkerboard pattern with an isoluminant commercial cartoon, thus serving as a reference baseline (Fig. 1 and Fig. S1). We found a significant increase of THb and OHb, with a parallel reduction of DHb concentration, in response to S appearance, reflecting the functional activation of visual areas in this condition as well. The cortical response was independent from the cartoon employed as baseline: a comparable HDR, indeed, was clearly elicited both when the baseline movie was fixed a priori by the experimenter (Cartoon Fixed condition, CF; Fig. 2B, Table S1) and when the cartoon was freely selected by the tested subject (Cartoon Chosen condition, CC; Fig. 2C, Table S1). Interestingly, a significant pattern of correlations emerged among HDR metrics recorded with different stimulating conditions (Fig. S2; see Table S1 for statistical details), indicating that the quality of visual input does not quantitatively impact HDR. The amplitude of cortical activation was only slightly smaller in response to CF and CC, with the range of OHb and DHb fluctuations being significantly lower with respect to that evoked by RS (Fig. 2D; Table S1).

Within the CF condition, we also established that the baseline cartoon does not affect the degree of visual activation: indeed, a comparable modification of THb, OHb, and DHb concentrations was recorded using “The Lion King”, “The Powerpuff Girls”, “Peppa Pig” or “Kung Fu Panda” (Fig. S3A; Table S1). Furthermore, no differences of visually evoked responses were detected modulating the contrast level of the baseline cartoon: THb, OHb, and DHb fluctuations, indeed, were comparable when a fixed baseline cartoon was presented at 20, 40, or 80% of contrast (Fig. 2E; Fig. S3B–D; Table S1). Finally, the response latency was homogenous in RS, CF, and CC conditions (Fig. S4A; Table S1).

Altogether, these results demonstrate the validity of this innovative stimulation procedure to evoke a significant and reliable response in the occipital cortex preserving inter-subject variability.

### The cartoon paradigm was reliable in eliciting cortical responses in children

We measured cortical responses in typically developing children (see Table 2 for demographics) viewing the radial checkerboard blended with the animated cartoon. We compared three different conditions: each subject, indeed, was asked to select two cartoons of their preference for the baseline, and the first choice was employed for the low-contrast (cartoon 1 low contrast, 20%, L1) and the high-contrast (cartoon 1 high contrast, 80%, H1) stimulation, while the second cartoon was presented only at low-contrast (cartoon 2 low contrast, L2; Fig. S1).

**Table 2:** Demographic characteristics of children.

ID	Age	Gender	Head	Cap size	AQ	AQ-S	AQ_C	AQ_A	AQ_D	AQ_I	C1 (L1 and H1)	C2 (L2)
B1	5	M	51.5	52	21	1	4	7	5	4	Uncle Grandpa S3E04 “Uncle Easter”, Cartoon Network Studios, 2016	Teen Titans Go! To the Movies Warner Bros. Animation, 2018
B2	5	M	51	52	32	6	7	3	10	6	Wile E. Coyote & Road Runner “Coyote falls”,”“Fur of flying” and “Rabid rider” episodes, Acme Corporation, 2010	ABCs song Little Baby Bum - Nursery Rhymes & Kids Songs youtube channel, 2014
B3	13	M	57	56	26	6	3	4	7	6	The Simpsons S29E02 “Springfield Splendor”, 20th Television, 2017	Futurama, S7E01 “The Bots and the Bees”, Comedy Central, 2012
B4	12	M	56	56	44	8	8	11	13	4	The Incredibles Disney-Pixar, 2004	Wile E. Coyote & Road Runner “Coyote falls”,”“Fur of flying” and “Rabid rider” episodes, Acme Corporation, 2010
B5	12	M	56	56	25	1	4	10	0	10	Big Hero 6 Walt Disney Pictures, 2014	The Amazing World of Gumball S01E01 “The DVD”, Cartoon Network Development Studio Europe, 2011
B6	6	F	53	52	17	1	1	4	8	3	Inside out Disney-Pixar, 2015	Floopaloo S1E22 “Squirrel for a Day”, Marc du Pontavice, 2012
B7	7	M	53	52	24	2	4	1	12	5	The Chipmunks “Bye, George” episode, DIC Entertainment, 1989	Floopaloo S1E22 “Squirrel for a Day”, Marc du Pontavice, 2012
B8	4	F	50.5	52	38	6	11	7	11	3	Frozen Walt Disney Pictures, 2013	Bolt Walt Disney Pictures, 2008
B9	4	M	52	52	42	1	11	11	13	6	Spider-Man: Into the Spider-Verse Columbia Pictures, 2018	Bolt Walt Disney Pictures, 2008
B10	4	M	51	52	n.a.	n.a.	n.a.	n.a.	n.a.	n.a.	Cars Walt Disney Pictures, 2006	Thomas & Friends “Diesel and the duckling” episode, Mattel, 2019
B11	9	F	54	56	28	2	6	3	13	4	The Simpsons S29E02 “Springfield Splendor”, 20th Television, 2017	Zig & Sharko S01E28 “Moby Zig”, Xilam Animation, 2010
B12	8	M	54.5	56	26	4	5	5	4	8	Beauty and the Beast Walt Disney Pictures,1994	Ice Age Blue Sky Studios, 2002
B13	4	M	53	56	44	11	9	8	9	7	Finding Nemo Disney-Pixar 2003	101 Dalmatians Walt Disney Productions,1961
B14	6	M	53	52	36	5	2	3	19	7	Trolls DreamWorks Animation, 2016	Curious George S1E03 “Zeros to Donuts”, Universal Animation Studios, 2006
B15	7	M	51.5	52	49	12	8	10	9	10	Cars Walt Disney Pictures, 2006	Pup Academy S1E02 “Tell Us About Your Human Day”, Air Bud Entertainment, 2019
B16	10	F	55	56	48	9	9	10	10	10	Frozen Walt Disney Pictures, 2013	Descendants Disney Channel Original Productions, 2015
B17	10	M	54	56	40	9	5	7	13	6	The Simpsons S29E02 “Springfield Splendor”, 20th Television, 2017	Uncle Grandpa S3E04 “Uncle Easter”, Cartoon Network Studios, 2016
B18	4	F	49.5	52	19	2	3	5	6	3	Frozen Walt Disney Pictures, 2013	Bing S1E06 “Smoothie”, Tandem Films e Digitales Studios, 2014
B19	7	M	54	56	19	2	3	5	6	3	Ranger Rob S1E12 “Big Stink in Big Sky Park” Nelvana Enterprises Inc, 2016	Arex&Vastatore “Casket of fear” episode Arex&Vastatore YouTube Channel, 2021

Age (years), gender, head circumference (head, cm), cap size (cm), total AQ score (AQ), AQ subscale scores (AQ_S, AQ_C, AQ_A, AQ_D, AQ_I), and the movies used for visual stimulation (C1 and C2 according to the experimental protocol) are listed for each participant. For movies, production company, release date, and episode title are indicated as well.

**Fig. 3:**
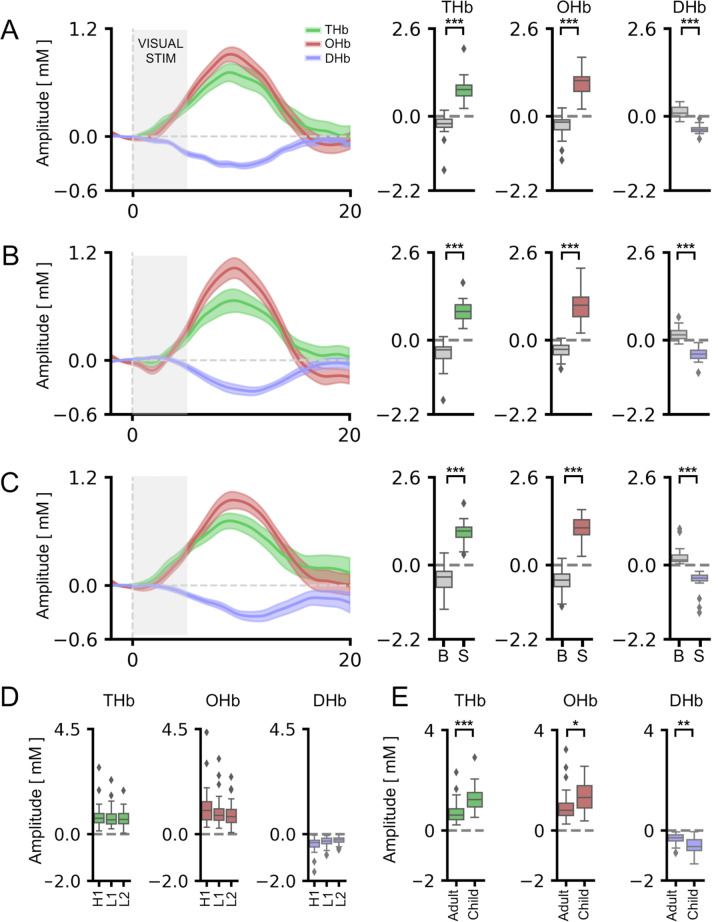
HDR signal was reliably detected in children using blended RS-animated cartoons with low and high contrast. For all panels, values in the y-axis are multiplied for 10^4. **A** On the left, the average time course for THb (green line), OHb (red line), and DHb (blue line) in response to high-contrast (80%) blended RS-animated cartoons is shown (CH1). On the right, the graphs represent the average amplitude of the evoked HDR following the stimulus (S) and the blank (B). A significantly different response to S with respect to the B was detectable for all metrics (t-test, *p* < 0.001 for all comparisons). **B** The average time course of the HDR to low-contrast (20%) blended RS-animated cartoons is shown (CL1). Here, the baseline cartoon is the same as the experiment described in panel (A), but a different part of the movie was used. THb, OHb, and DHb showed a significantly higher deflection to the S with respect to the B in this condition as well (t-test, *p* < 0.001 for all comparisons). **C** On the left, the average time course of HDR following the second low-contrast blended RS-animated cartoon selected by the subject (CL2). On the right, the analysis of peak amplitudes revealed significantly higher responses during S compared to B for THb, OHb, and DHb (t-test, *p* < 0.001 for all comparisons). **D** Comparison among different contrast levels of the baseline cartoon revealed no differences in the amplitude of HDR. **E** Response amplitudes for low-contrast blended RS-animated cartoons in adults and children. More specifically, we compared the response to CF condition of adults with CL1 condition for children. The average amplitude of HDR was significantly higher in children (t-test, *p* < 0.001 for THb, *p* < 0.05 for OHb, *p* < 0.01 for DHb). For statistical metrics and details, refer to Table S1. Data are shown as average ± s.e.m. * *p* < 0.05; ** *p* < 0.01; *** *p* < 0.001.

Our data showed a significant activation of the visual cortex, with a prominent change of THb, OHb, and DHb concentration in response to the S with respect to the blank for all conditions tested (Fig. 3A–C; see Table S1 for statistical details). The amplitude of elicited cortical responses was comparable following L1, H1, and L2 (Fig. 3D; Table S1), proving that the HDR is independent from the cartoon narrative selected for the baseline and the contrast level of baseline presentation in children as well. Small differences were observed for response latency among L1, H1, and L2 conditions (Fig. S4B; Table S1). A highly significant pattern of correlations among different HDR indexes recorded in the diverse conditions was detected (Fig. S5; see Table S1 for statistical details).

In agreement with previous literature [79], a maturational trend of cortical responsivity was recognized, with children showing significantly higher HDR amplitude with respect to adult subjects (Fig. 3E; Table S1). On the contrary, age-dependent effects were not identified for response latency (Fig. S4C; Table S1).

These findings establish a novel method for measuring visually-evoked cortical activity with fNIRS that ensures an elevated compliance of young subjects and high-quality reliability of measurements, suggesting a valuable tool for studying visual cortical processing in typically developing children, but also in clinically relevant populations.

### Negative correlation of HDR amplitude with AQ score

**Fig. 4:**
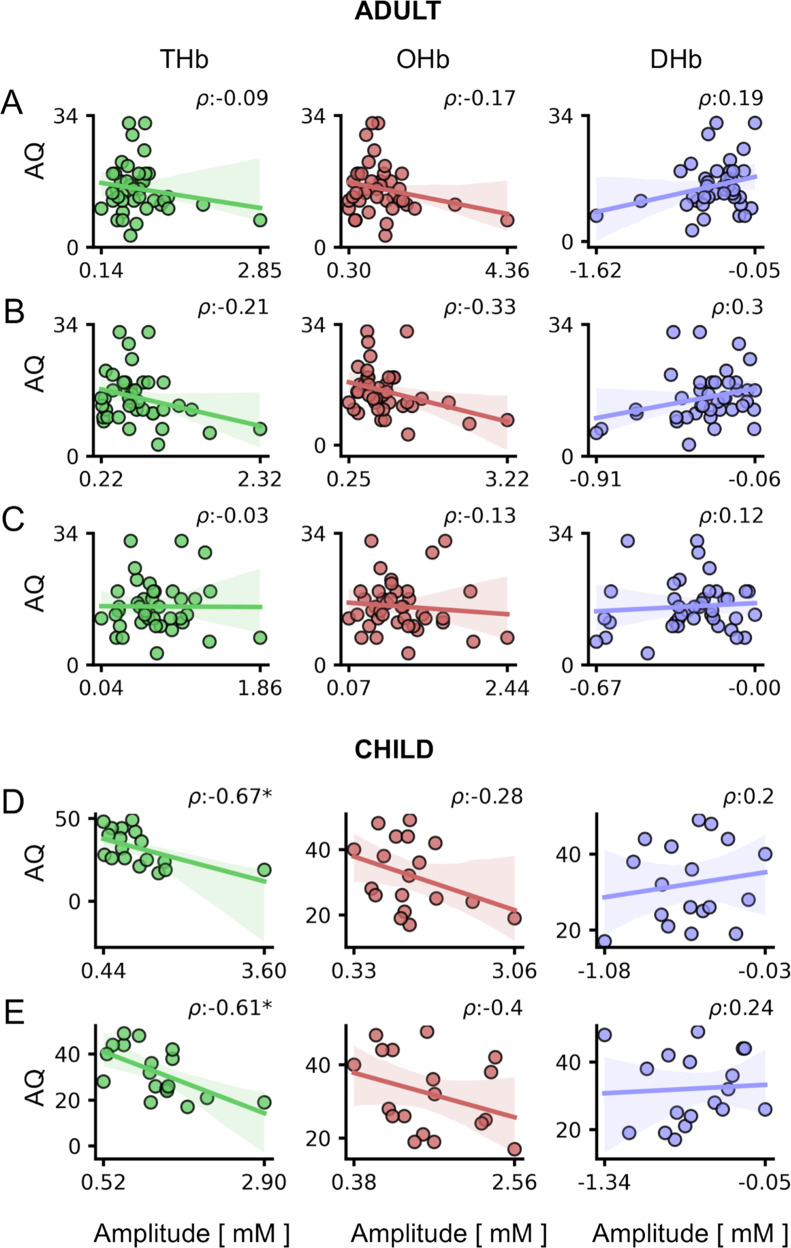
Correlation between HDR and AQ scores. For all panels, values in the *x*-axis are multiplied for 10^4. The *ρ* (rho) index in each plot indicates the Spearman correlation value. Correlation between HDR and AQ scores in adults, for amplitudes obtained using RS (**A**), CF (**B**), and CC (**C**). No significant correlations were detected for adult participants. Correlation between HDR and AQ scores in children, for amplitudes obtained using high (**D**), and low (**E**) contrast baseline cartoons. A significant correlation was found between THb and AQ scores for both high- and low-contrast blended stimuli (*p* < 0.05 for both cases). Circles are individual values; lines represent the linear regression model fit and shaded regions are the 95% CI.

Despite no effects detectable in adults (Fig. 4A–C; Table S1), the amplitude of visual responses was highly correlated to AQ scores in children (Fig. 4D, E; see Table S1 for statistical details). Consistent with a recent work [57], the correlation was specific for THb, with higher AQ score being associated with a lower amplitude of THb visually-evoked signals (Fig. 4D, E).

**Fig. 5:**
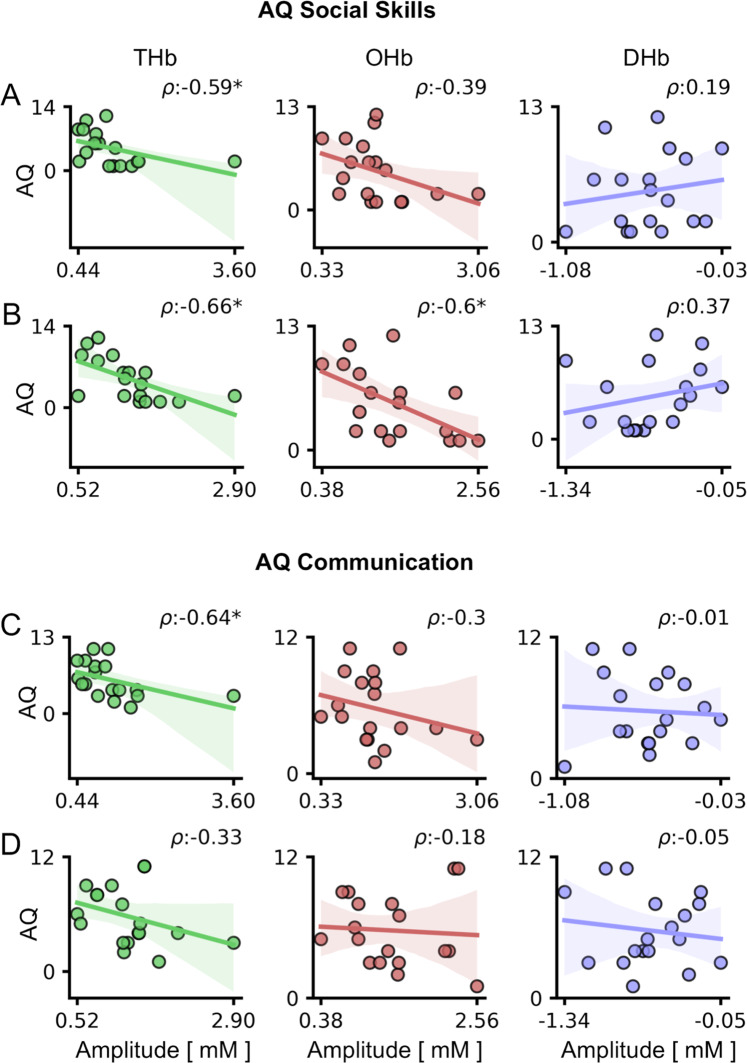
Correlation between HDR and AQ subscales in children. For all panels, values in the *x*-axis are multiplied for ×10^4. The *ρ* (rho) index in each plot indicates the Spearman correlation value. **A**, **B** Correlations between HDR and AQ Social Skills (AQ_S) subscale. A significant correlation between THb and AQ_S was detected using both high- (**A**) and low-contrast blended stimuli (**B**; *p* < 0.05 for both cases). In addition, OHb recorded in response to the low-contrast blended RS-cartoon was significantly correlated with AQ_S (**B**; *p* < 0.05). **C**, **D** Correlations between HDR and AQ Communication (AQ_C) subscale. THb amplitude in response to the high-contrast blended RS-cartoon was significantly correlated with AQ_C (*p* < 0.05). Circles are individual values; lines represent the linear regression model fit and shaded regions are the 95% CI.

Interestingly, HDR amplitude was especially linked to social and communication autistic traits (Fig. 5, S6; Table S1): indeed, assessing separately the five AQ subscales [77] we found a significant correlation of THb and OHb with the Social Skills subscale (AQ_S, Fig. 5A, B), while only THb modulation was related to the Communication subscale (AQ_C, Fig. 5C, D) and no significant interaction was observed testing the other three AQ subscales (AQ_A, AQ_D, and AQ_I; Fig. S7). Given the reliability across different visual tasks (L1 and H1), the strongest interaction was between THb and AQ_S (Fig. 5A, B).

## Discussion

We measured hemodynamic responses in the occipital cortex while subjects viewed a reversing checkerboard pattern on a gray isoluminant baseline or the same stimulus blended with a commercial animated cartoon. In all participants, the patterned stimulus elicited a significant change of cortical Hb (upwards for THb and OHb, down for DHb) independently from the reference baseline, while no response was detected following blank presentation. Since HDR consists of an initial increase in oxygen-rich blood followed by a smaller depletion of deoxy-hemoglobin, with a tight interrelation among total, oxygenated, and reduced Hb levels, reporting all data allows for a more accurate physiological interpretation of the results [78].

Interestingly, the level of occipital cortex activation did not depend on either the movie selected as reference baseline or the baseline contrast, with only a slight reduction of OHb and DHb change in response to the stimulus blended to animated cartoons with respect to the classic RS condition. These data demonstrate the reliability of this novel procedure with high entertaining and ecological value in eliciting cortical activity. Thus, our approach might be helpful for studying cortical function in children with an atypical trajectory of brain development, commonly showing a reduced compliance in experimental environments.

The magnitude of HDR modulation, and in particular of THb, was inversely correlated with AQ scores in children. Indeed, we found that the higher were the AQ scores of subjects the lower was the amplitude of THb response to visual stimulation, suggesting that visually-evoked fNIRS responses are able to capture the dimension of autistic traits in the general young population. Our findings are consistent with previous studies showing that cortical activation measured with fNIRS and the performance in visual psycophysics negatively reflected ASD symptom severity [45, 46, 71, 72, 80–83]. Moreover, these data reinforce the concept that THb changes could provide richer discriminative information for classifying between typically developing children and ASD subjects [57]. In our experiment, the THb index describes about 45% of the variance in AQ scores. This correlation is remarkably high, considering that it is detected across two separate visual stimulating procedures, i.e., the vision of low- and the high-contrast blended RS-animated cartoons. Since levels of THb reflect the relative changes of both OHb and DHb concentrations over the visual cortex [21], we surmise that the better correlation of THb with AQ scores might be due to a combinatory effect of OHb and DHb variables. The information about OHb increase and DHb reduction for each subject, indeed, converges in the modification of THb levels [78].

A tentative explanation of reduced HDR in children with stronger autistic traits might be found in the difference of perceptual styles in the general population [84, 85]: the preference for focusing on local details vs. the global stimulus configuration, indeed, is a defining feature of ASD [86] and locally centered perception could be less effective in activating the neural circuits of visual cortex. Interestingly, a recent study established a vascular link to ASD, showing early dysfunction of endothelial cells and impaired endothelium-dependent vasodilation in a mouse model of 16p11.2 deletion [87]. Since the HDR measured with fNIRS strongly relies on neurovascular coupling, this suggests that lower neurophysiological activity may stem in part from endothelial-dependent vascular factors.

In contrast, we failed to detect any correlation between HDR and AQ scores in adult subjects. This is likely due to the different output range of variables measured in children and adult participants. Accordingly, the 4-point Likert scale used for questionnaires in child population changes the range of AQ scores from 0–50 to 0–150, potentially revealing a different variability of inter-individual traits. Moreover, the maturation of neural and vascular networks over brain development affects the pattern of hemodynamic responses [88, 89]. Indeed, our data showed that the amplitude of fNIRS visually-evoked responses is significantly higher in children compared to adults, with a parallel broader distribution of recorded signals.

Although the autistic questionnaire for children is well-validated, showing good test-retest reliability and high internal consistency, not all items have the same validity and factor analysis identified five subscales, named ‘Social Skills’, ‘Communication’, ‘Attention to Detail’, ‘Imagination’ and ‘Attention Switching’ [77]. We observed variability in the strength of correlation between HDR and AQ subscales, with the highest correlation for the ‘Social Skills’ and ‘Communication’ subscales. Interestingly, ‘Social Skills’ and ‘Communication’ are the subscales with higher construct validity performance in differentiating individuals with or without ASD [90, 91], while ‘Attention to Detail’ was the poorest classifying domain [92]. It is also worth stressing that among the items composing the ‘Attention to Detail’ subscale, only four actually relate to sensory perception (e.g., ‘My child usually notices details that others do not’), the others being more focused on cognitive functions (e.g., ‘My child is fascinated by numbers’). Accordingly, the first pilot report of the AQ questionnaire showed lower internal consistency (measured by Cronbach’s alpha coefficient) of ‘Attention to Detail’ items compared to other subscales [76].

Although primarily affecting social functioning, there is a growing body of evidence showing that ASD is also associated with abnormalities in multiple sensory domains, fluctuating between hyper- and hypo-sensitivity to sensory stimuli [80, 93]. In addition to a higher incidence of refractive errors and strabismus [94], anomalies in visual processing, visual attention, and visual-motor integration have been described in ASD population [71, 72, 74, 80, 95]. Interestingly, sensory symptoms are correlated with the severity of the disorder, at least in children [96]. Moreover, commonly observed alterations in social skills might have a visual component [80] and perception deficits could impact with cascading effects on the maturation of cognitive and social domains [95].

It has been recently suggested that an early assessment of pupil size modulation and visual behavior might improve the diagnostic process of ASD [74, 84, 95, 97]. Currently, ASD diagnosis and follow-up almost entirely rely on phenotypic information collected via clinical measures and parental input that are highly prone to subjective bias [98]. Moreover, the late appearance of some behavioral autistic traits often delays the diagnosis until mid-childhood [99, 100]. Thus, the identification of solid brain biomarkers early predicting ASD pathophysiology is a critical step to anticipate tailored interventions, leading to better outcomes for patients and possibly even the prevention of certain behaviors typically associated with ASD. Objective biomarkers have also the potential to be helpful in the management of patients, allowing the classification of disease severity and monitoring response to treatments [14]. A recent systematic review highlighted that both functional and structural neuroimaging features might predict ASD diagnosis in the early pre-symptomatic period [101, 102], but further studies are needed to validate the promising performance of such biomarkers [14]. Lately, resting-state fNIRS measurements have been suggested as candidate biomarkers for ASD [32, 57, 59, 60]. As stressed above, fNIRS offers significant advantages with respect to other neuroimaging tools, including non-invasiveness, ease of use, no need of sedation, tolerance to movements, and portability, making it a child-friendly approach. However, the extraction of metrics with diagnostic value from resting-state recordings involves complex algorithms.

In contrast, our analysis of visually evoked responses is quick, easy, and requires only that children pay attention to a short movie of their choice. Since our stimulating strategy has been studied to optimize the compliance of young subjects, we believe that our results might set the background for testing fNIRS visual measurements in ASD individuals. Moreover, screening for autistic traits in the general population may be helpful in epidemiological research because it may provide a large sample size to investigate the correlation between autism phenotype severity and other pathophysiological processes [91].

Although our study provides a first step towards the use of fNIRS for empowering early detection of autistic traits, some limitations need to be discussed. First, the quantification of AQ scores for children according to parent questionnaires might introduce a response bias in the dataset [103]. Despite the high test-retest and reliability coefficients of AQ-Child [77], future studies might examine autistic traits in the child general population with an integrated approach consisting of direct behavioral observation and administration of multidimensional questionnaires. Moreover, testing a larger and gender-balanced sample will allow not only to confirm the validity of our results, but also to potentially highlight gender differences in fNIRS measurements and to stratify the child population in different age groups. Finally, combined fNIRS-EEG recordings are needed to dissect whether the sensitivity of fNIRS to autistic traits is determined by neural or vascular processes.

## Materials and methods

### Subjects

We recruited a total of 40 adult subjects (20 women, age: 31.05 ± 3.94 (SD) years) and 19 children (5 girls, age: 7.20 ± 3.01 (SD) years). All participants reported normal or corrected-to-normal vision and had no diagnosed neuropsychiatric condition. Experimental procedures on children were authorized by the Regional Pediatrics Ethics Board (Comitato Etico Pediatrico Regionale-Azienda Ospedaliero-Universitaria Meyer-Firenze, Italy; authorization number 201/2019) and were performed according to the declaration of Helsinki. Written informed consent was obtained from all adult participants and from the parents of each child, authorizing the use of anonymized data for research purposes. Assent was also obtained from the children involved in the study before participation.

### AQ score

Adult participants filled in the Autistic-Spectrum Quotient (AQ) questionnaire, a 50-items self-administered report validated for the Italian version [76, 104]. The items consist of descriptive statements assessing personal preferences and typical behavior. For each item, participants respond on a 4-point Likert scale: “strongly agree”, “slightly agree”, “slightly disagree”, and “strongly disagree”. The items are grouped in five subscales: Social Skills, Communication, Attention to Details, Imagination, and Attention Switching. All the questionnaires were scored by a neuropsychiatrist blinded to subject data: 1 point was assigned when the participant’s response was characteristic of ASD (slightly or strongly), 0 points were attributed otherwise. Total scores range between 0 and 50 (0–10 for each subscale), with 32 being the clinical threshold for autism risk [76]. No subjects scoring above 32 points were recorded. The mean (min-max) of the scores was 15.1 (3–32) with SD of 6.5 (Table S2). The children’s version of Autism-Spectrum Quotient (Italian version of AQ-child) was completed by parents [77]. This version of the AQ questionnaire includes 50 items as well, grouped in the same subscales described above, and parents were required to report for each statement the degree of consistency with their child’s behavior. Scores range from 0 to 150 (0–30 for each subscale), since the response scale is treated as a 4-point Likert scale with 0 representing definitely agree; 1 slightly agree; 2 slightly disagree; and 3 definitely disagree. Items were reverse-scored as needed. The threshold score is 76 [77]. All subjects scored below 76 points. The mean (min-max) of the scores was 32.1 (17–49) with SD of 10.7 (Table S3).

### Apparatus and montages

To measure changes in total Hb (THb) concentration and relative oxygenation levels (OHb and DHb) in the occipital cortex during the task, we used a continuous-wave NIRS system (NIRSport 8×8, NIRx Medical Technologies LLC, Berlin, Germany). Our NIRSport system consists of eight red light-sources operating at 760 and 850 nm, and seven detectors that can be placed into a textile EEG cap (EASYCAP, Herrsching, Germany), forming an array of 22 multi-distant channels [105]. Textile EEG caps of different sizes were used. The probe arrangement was fixed in each of the caps using grommets, optode stabilizers, colored labels, and holders in order to assure comparable probe mapping over all subjects. For data recording, the Aurora Software 1.4.1.1 (NIRx Medical Technologies LLC) was employed. The sampling rate was 10.2 Hz. Visual areas were identified according to the craniocerebral topography within the international 10-20 system and the placement of the optodes was done using fOLD v2.2 [106] and NIRSite 2.0 (NIRx Medical Technologies LLC) softwares. Sources and detectors were symmetrically distributed to define 22 channels around the region of interest, each adjacent pair of sources and detectors defining one channel (min-max source-detector separation: 20–44 mm for adults, 22–30 mm for children; Fig. S2).

### Experimental design and visual stimulation

Prior to the experiment, adult participants (or parents for children) filled in the AQ questionnaire. Then, subjects were asked to sit on a comfortable chair and the fNIRS cap was positioned. Optodes were placed into the cap and the calibration of light coupling between sensors and detectors was performed. All experimental sessions lasted 30–40 min. Visual stimuli were generated using Python 3 and Psychopy3 [107] and displayed with gamma correction on a monitor (Sharp LC-32LE352EWH, 60 Hz refresh rate, 45 cd/m^2^ mean luminance, resolution of 800×600 pixels) placed 70 cm from the subject. Cortical hemodynamics in response to full-field, reversing, square wave, radial checkerboard, with abrupt phase inversion (spatial frequency: 0.33 cycles per degree, temporal frequency: 4 Hz; Fig. 1A) was evaluated in the time domain by measuring the peak-to-baseline amplitude and latency. To have an internal control with blank stimulation, we used an event-related design consisting of: (i) 20 cycles of 5 s stimulus ‘on’ (reversing checkerboard, 90% of contrast) followed by 10 s stimulus ‘off’ and (ii) 20 cycles of 5 s mock stimulus ‘on’ (reversing checkerboard, 0% of contrast) followed by 10 s stimulus ‘off’. The two stimulating conditions were pseudo randomly interleaved for each subject during the recording. Blocks lasted 10 min and participants were permitted to take rest between recordings. Figure 1D shows a schematic representation of the experimental procedure. Visual events were synchronized with NIRSport over wireless LAN communication through the Python version of LabStreamingLayer (https://github.com/sccn/labstreaminglayer).

#### Recordings in adult participants

Experiment 1 for adults (exp1) aimed to understand whether a reliable hemodynamic signal could be recorded in response to the radial checkerboard merged with an animated cartoon. Thus, exp1 started with a 10 min recording using the reversing checkerboard as stimulus ‘on’ and the gray screen as stimulus ‘off’ (RS condition), and continued with the vision of two different blended animated cartoons, where the stimulus ‘on’ was a merge between the reversing checkerboard and the movie, whereas the stimulus ‘off’ was the gray-scale isoluminant cartoon (CF and CC conditions; Fig. S1). The main purpose of using a cartoon was to increase the entertaining value of visual stimulation. The checkerboard presentation was needed to ensure a standardized episodic stimulation allowing event-related transient analysis. We decided to merge the checkerboard with the movie in order to avoid possibly distracting interruptions of the storyline and to facilitate screen fixation in children. The merging procedure was achieved using Python3 OpenCV [108]. During the appearance of the stimulus each frame was filtered using an automatic Canny edge detection algorithm (https://www.pyimagesearch.com/2015/04/06/zero-parameter-automatic-canny-edge-detection-with-python-and-opencv/), then the filtered cartoon was blended with the radial checkerboard. Each pixel of the animated cartoon with the same color of the corresponding pixel of the radial checkerboard was inverted, to obtain a fully visible image. The result was a RS with an overlayed cartoon frame (Fig. 1B). The first cartoon was randomly selected by the operator within a group of 4 (“The Lion King”, “The Powerpuff Girls”, “Peppa Pig” or “Kung Fu Panda”; CF), whereas the latter was a free choice of the subject (CC). Exp2, aiming to dissect the contribution of baseline contrast to visual responses, was performed in a subset of adult participants (*n* = 15). Exp2 consisted of 3 consecutive recordings of a CF (“Peppa Pig”; “Hide-and-seek”, “Fly the kite”, “Polly parrot” episodes) with the modulation of the baseline contrast (20, 40, and 80%; Fig. S1). The presentation order of different contrast levels was randomly shuffled.

#### Recordings in children

To confirm that the baseline movie and its contrast do not affect the emergence of visual responses to the radial checkerboard in children, we measured hemodynamic signals in response to 2 different blended RS-animated cartoons freely decided by the subject: cartoon 1 was presented at both low (20%, L1) and high (80%, H1) contrast, while only low contrast was recorded for cartoon 2 (L2; Fig. S1). In this case, the presentation order was decided by the child, in order to maximize subject compliance.

During the experimental sessions, data were quickly analyzed and visualized using nirsLAB software (NIRx Medical Technologies LLC, v2019.4).

### Signal processing and statistical analysis

Data preprocessing was completed using the Homer3 package (v1.29.8) in MATLAB (R2020a). We created a processing stream tailored on recent guidelines for the analysis of fNIRS data [25]. First, the raw intensity data were converted to optical density (OD) changes (*hmR_Intensity2OD*). Then, channels showing very high or low optical intensity were excluded from further analyses using the function *hmR_PruneChannels* (dRange: 5e-04-1e+00, SNRthresh: 2; SDrange: 0.0–45.0). Motion artifacts were then removed by a multistep rejection protocol. After a step of motion artifact detection using the *hmR_MotionArtifactByChannel* function (tMotion: 1.0, tMask: 1.0; STDEVthresh 13.0; AMPthresh: 0.40), motion correction was performed with a combination of Spline interpolation (*hmR_MotionCorrectSpline*, p: 0.99) and Wavelet filtering (*hmR_MotionCorrectWavelet*, iqr: 0.80) functions [25]. The remaining uncorrected motion artifacts were identified using the *hmR_MotionArtifactByChannel*. A band-pass filter (*hmR_BandpassFilt: Bandpass_Filter_OpticalDensity*, hpf: 0.01, lpf: 0.50) was applied to decrease slow drifts and high-frequency noise, and the OD data were converted to Hb concentration changes using the modified Beer–Lambert law (*hmR_OD2Conc*, ppf: 1.0 1.0 1.0). Finally, trials of each subject were block-averaged for every stimulating condition and channel (*hmR_BlockAvg: Block_Average_on_Concentration_Data*, trange: −2.0 20.0) [25]. The resulting txt file was imported in Python as a Pandas DataFrame. For each subject, only the channel with the highest response amplitude was analyzed. The peak response was identified as the maximal value for THb and OHb and the minimum value for DHb. A grand average was taken of the 20 trials of data per stimulating condition and differences between visual stimulation ‘on’ (reversing checkerboard) and ‘off’ (blank) were compared. All data were normalized with respect to the blank-evoked response using a subtraction method. Statistical analysis was carried out using *pingouin* Python library [109] and the following functions: *pingouin.ttest* (paired and two-sided t-test), *pingouin.rm_anova* (one-way repeated measures ANOVA), *pingouin.pairwise_ttests* (post hoc analysis), *pingouin.pairwise_corr* (Spearman correlation), *pingouin.regplot* (Linear regression). T-test, ANOVA, and post hoc analysis were used to assess differences in fNIRS peak responses following different stimulating conditions, whereas we tested the interaction between the amplitude of fNIRS measures and AQ scores with Spearman correlation. Total AQ scores, and AQ_S, AQ_C, AQ_A, AQ_D, and AQ_I subscale scores were used for correlational analysis. We employed the Linear regression to plot such correlations. For the correlational analysis adjustments for multiple comparisons were performed using the Benjamini/Hochberg false discovery rate (BH-FDR) correction. The effect size calculated for the ANOVA was the generalized eta-squared. All the plots have been generated using *Matplotlib* Python library [110]. All statistical metrics and details are reported in Supplementary Table 1 (Table S1).

## Supplementary information

Supplemental material

## Data Availability

The datasets generated during the current study and scripts used for visual stimulation are available, respectively on Zenodo (10.5281/zenodo.5101912) and GitHub website (https://github.com/raffaelemazziotti/FNIRS_code).
