# Supplementary material for: The amplitude of fNIRS hemodynamic response in the visual cortex unmasks autistic traits in typically developing children
Source: Transl Psychiatry. 2022 Feb 8;12:53. doi: 10.1038/s41398-022-01820-5 (PMC8826368; doi:10.1038/s41398-022-01820-5)
Supplement: Supplementary file 1 — Supplemental material [file 41398_2022_1820_MOESM1_ESM.pdf]

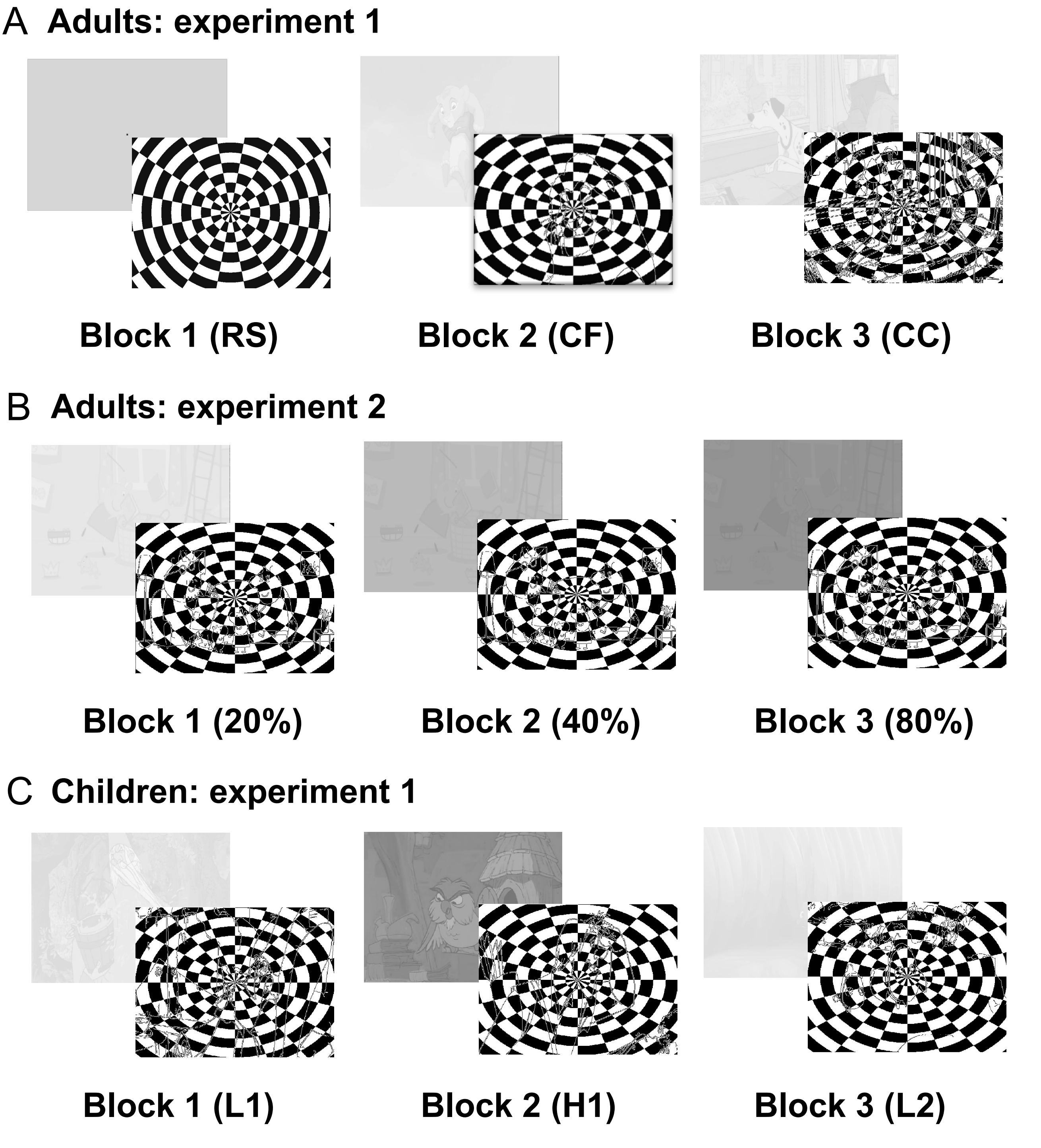

Fig. S1: Experimental plan. For each block (i.e., stimulating conditions), representative frames of baseline screen (stimulus 'off', up) and reversing checkerboard (stimulus 'on', low) were shown. **(A)** Experiment 1 for adult participants consisted in three 10-minute blocks, starting with the vision of the classic radial checkerboard-on-grey stimulus (RS condition) and continuing with two different RS-blended animated cartoons. The first cartoon was decided by the operator within a group of 4 (CF condition), whereas the latter was a free choice of the subject (CC condition). **(B)** Experiment 2 was carried out in a subset of adult participants (n = 15), watching the same «Peppa Pig» episodes with the modulation of the baseline contrast (20%, 40&, 80%). **(C)** Children were recorded during the vision of 2 different blended animated cartoons freely selected by the subject: cartoon 1 was shown at both low (20%, L1) and high (80%, H1) contrast, while only low contrast was used for cartoon 2 (L2).

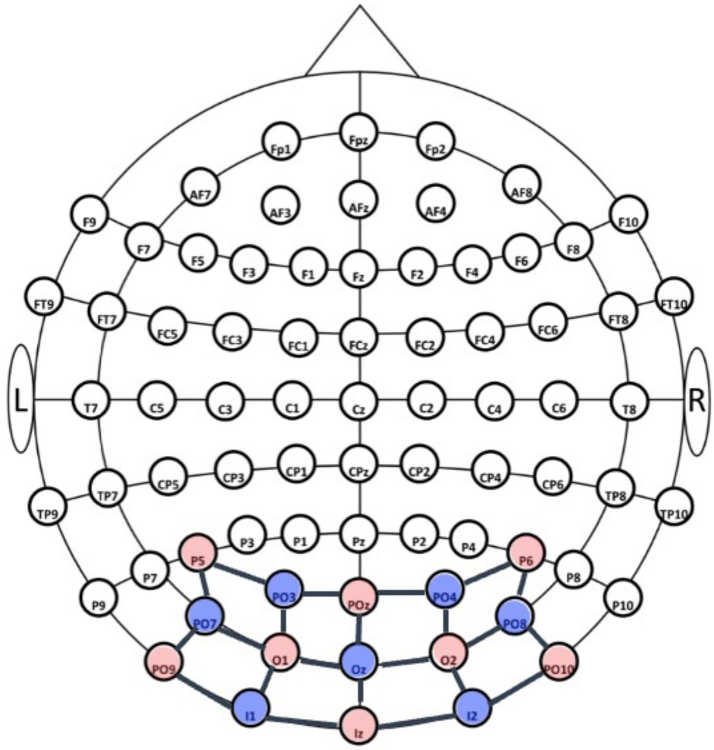

Fig. S2: Occipital 8x7 montage according to the international 10-20 system (the only differences in the correspondent adult occipital montage are: PPO5h for P5 and PPO6h for P6); sources (red circles) and detectors (blue circles) were symmetrically distributed to define 22 channels around the visual areas of the cortex, each adjacent pair of sources and detectors defining one channel.

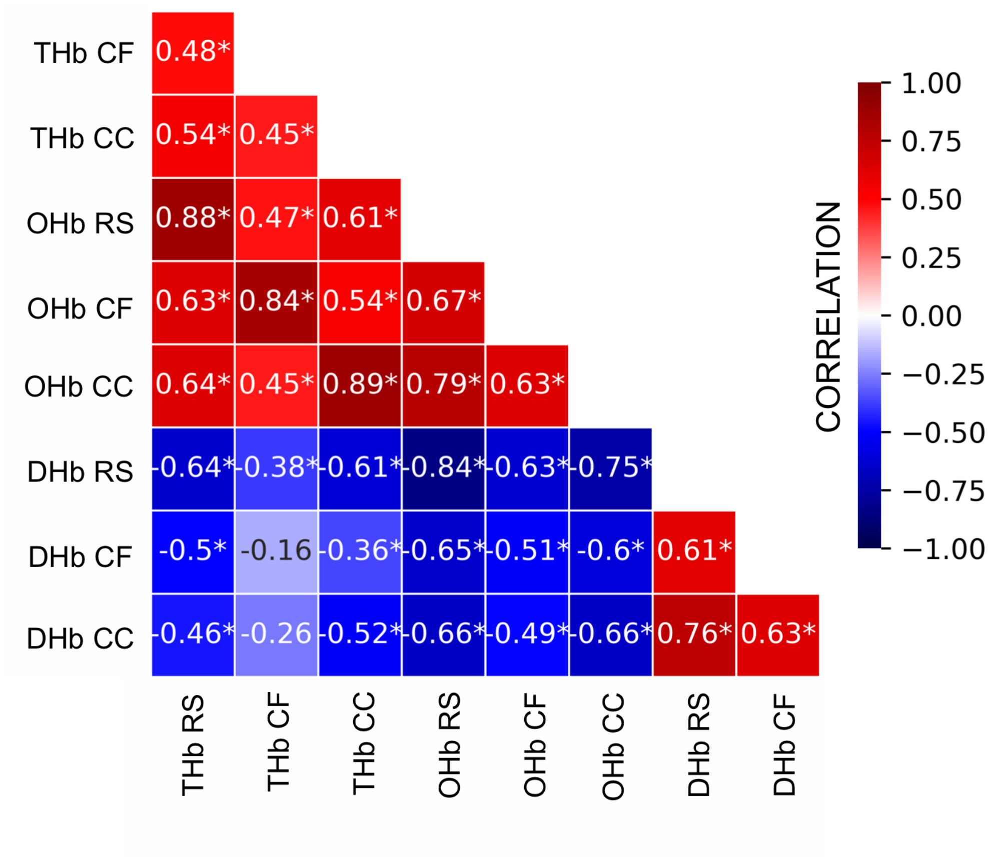

Fig. S3: Matrix of correlations among different HDR metrics recorded in response to the diverse visual stimulations presented to adult participants. A highly significant pattern of correlations emerged, indicating that stimulating conditions were largely interchangeable. Asterisks indicate significant correlations.

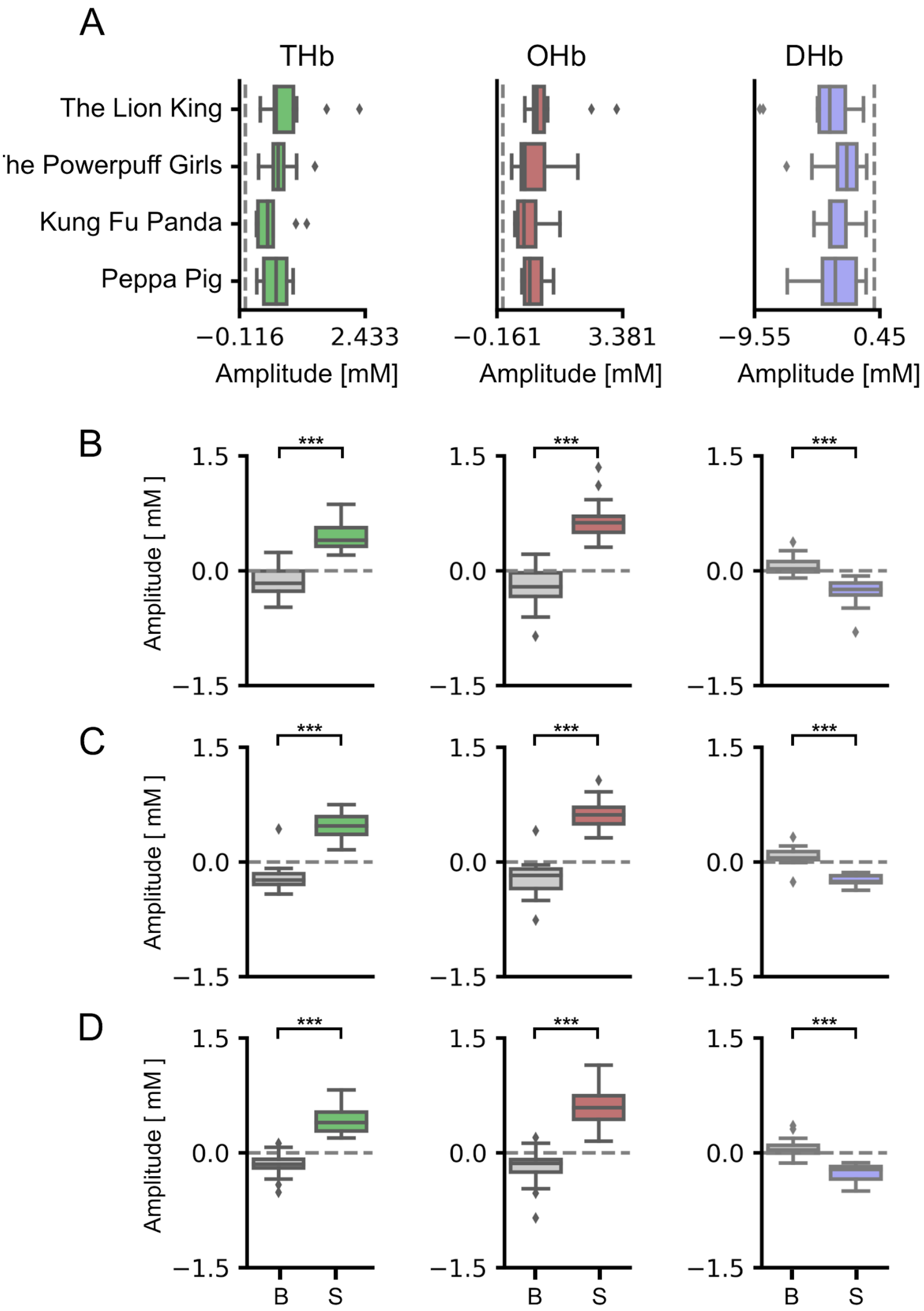

Fig. S4: The amplitude of evoked responses did not depend on either the cartoon selected as reference baseline or the contrast level of the movie. A: Values in the x-axis are multiplied for 10^4. No significant differences were detected in the cortical response elicited by RS blended with different baseline cartoons in adults. **B-D:** Comparison between the stimulus (S)-evoked amplitude against blank (B) using blended cartoons of low (B), medium (C) or high (D) contrast level in adults. The stimulus-evoked amplitude was significantly higher than that recorded during the B (t-test, $p < 0.001$ for all comparisons).

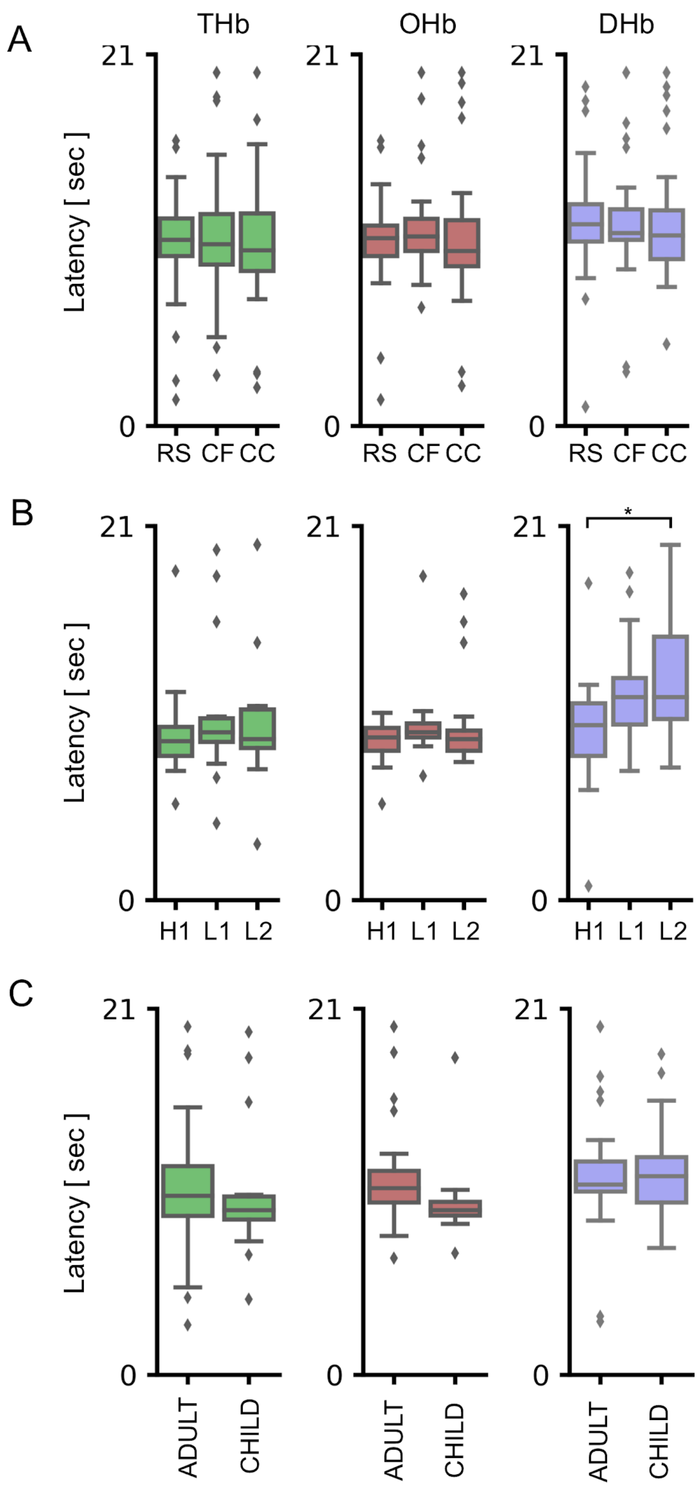

Fig. S5: Latency of HDR in adults and children. A: The analysis of latency to peak in adults revealed no differences among RS, CF and CC. **B:** In children, no differences of HDR latencies were found between H1 and L1, whereas a significantly higher latency of DHb was detected between H1 and L2 (One-way RM ANOVA, $p < 0.05$, post hoc BH-FDR H1 vs. L2 $p < 0.05$). **C:** No significant differences were identified for latencies between adults and children. * $p < 0.05$.

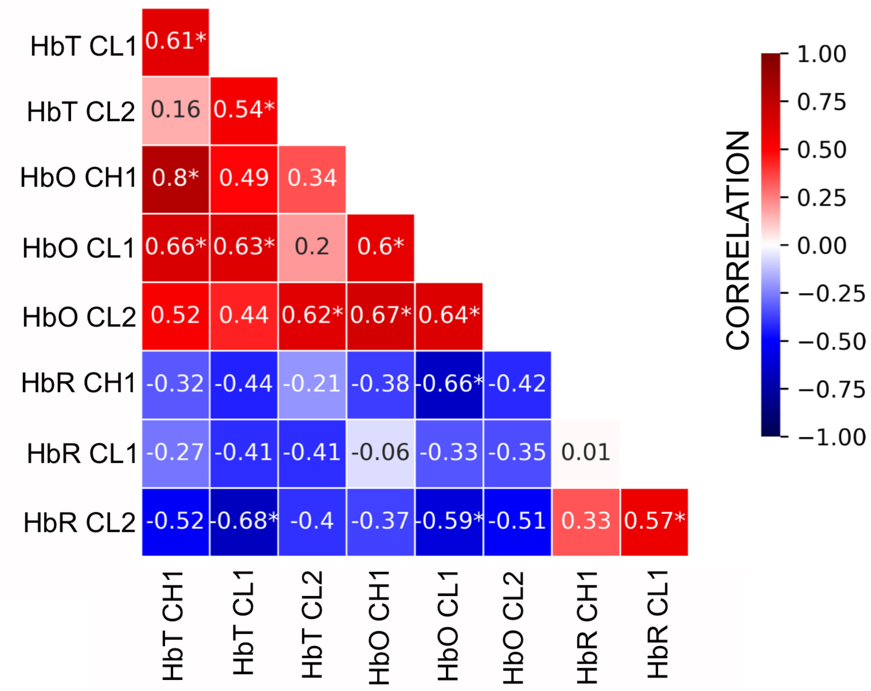

Fig. S6: Matrix of correlations among different HDR metrics recorded in response to the diverse visual stimulations presented to children. Similarly to the recordings in adults, a highly significant pattern of correlations was found.. Asterisks indicate significant correlations.

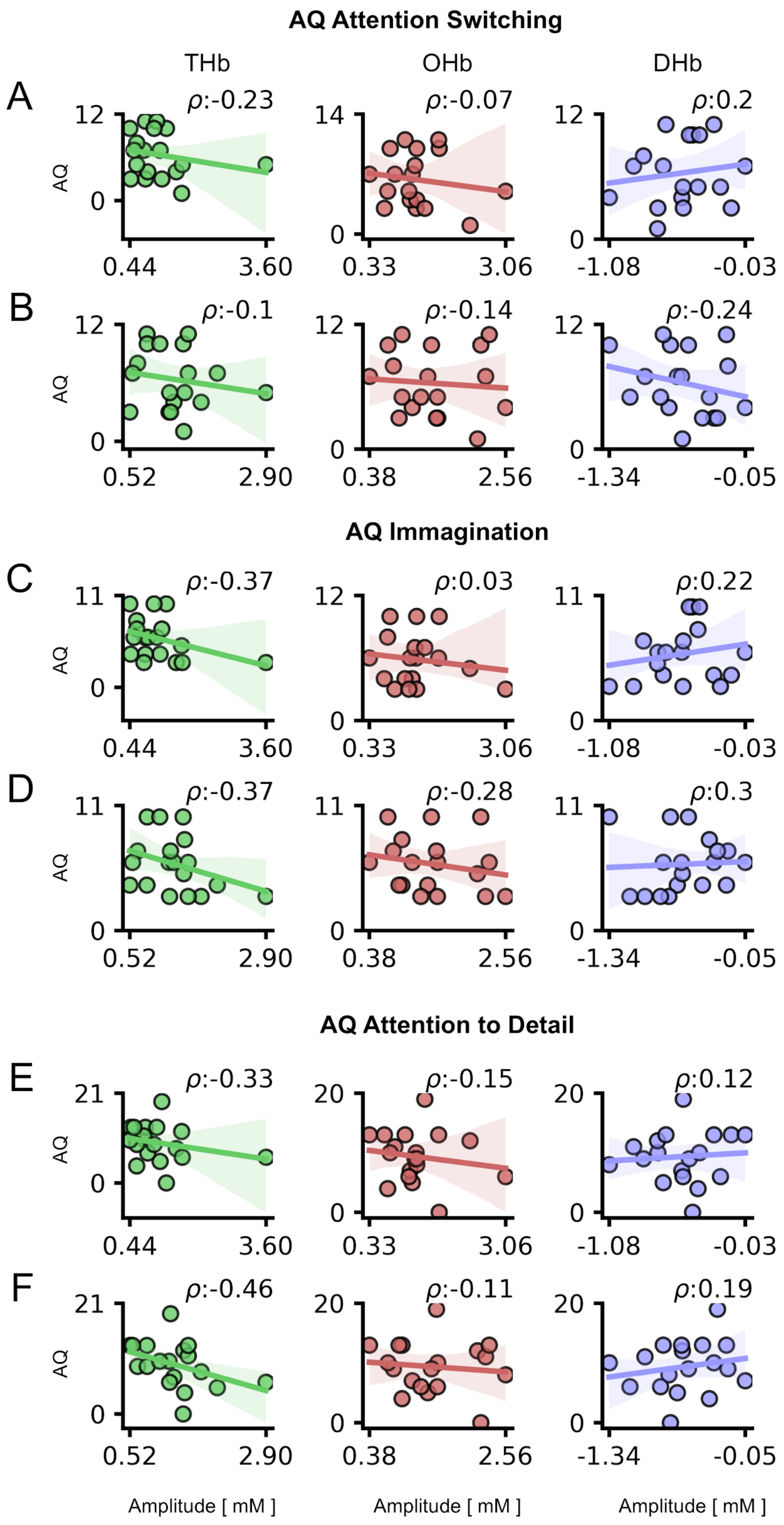

Fig. S7: Correlation between HDR and AQ subscales in children. For all panels, values in the x-axis are multiplied for 10^4. The ρ (rho) index in each plot indicates the Spearman correlation value. No significant correlation was present
Between THb, OHb, DHb metrics and Attention Switching (A,B), Imagination (C,D) and Attention to Detail (E,F) AQ subscales.

Supplementary Table 1 (Table S1). Statistical Table. **Hb**: Hemoglobin, **cond**: condition, **T**: t-distribution value, **power**: the achieved power of the test (1 - type II error), **BF10**: Bayes Factor of the alternative hypothesis, **p-val**: p-value (corrected for sphericity violation in anova rm and multiple comparisons in post hoc), **ng2**: generalized eta-squared, **eps**: Greenhouse-Geisser epsilon factor, **corr**: correction, i.e., multiple comparisons method, **fdr_bh**: Benjamini/Hochberg FDR correction, **r**: regression coefficient, **F**: F-statistic, **eta-square**: eta-squared effect size.

Figure	Hb	cond	test	T	tail	p-val	power	BF10		
2 A	THb	rs	T-test	-10.27664192	two-sided	1.17E-12	1	7.09E+09		
2 B	THb	cf	T-test	-10.53736721	two-sided	5.69E-13	1	1.42E+10		
2 C	THb	cc	T-test	-11.45204453	two-sided	4.80E-14	1	1.52E+11		
2 A	OHb	rs	T-test	-9.408641697	two-sided	1.40E-11	1	6.64E+08		
2 B	OHb	cf	T-test	-10.10992018	two-sided	1.88E-12	1	4.53E+09		
2 C	OHb	cc	T-test	-10.48477915	two-sided	6.58E-13	1	1.24E+10		
2 A	DHb	rs	T-test	9.473101868	two-sided	1.16E-11	1	7.94E+08		
2 B	DHb	cf	T-test	9.910922592	two-sided	3.29E-12	1	2.64E+09		
2 C	DHb	cc	T-test	9.799223827	two-sided	4.53E-12	1	1.95E+09		
**Figure**	**Hb**	**Test**	**F**	**p-val**	**ng2**	**eps**				
2 D	THb	anova_rm	1.640868453	0.2004303395	0.009952442677	0.9901442088				
2 D	OHb	anova_rm	7.340360394	0.001199783266	0.03338550065	0.8892069675				
		**post hoc**	**A**	**B**	**T**	**Tail**	**p-val**	**corr**	**eta-square**	**BF10**
		t-test	best_cc_delta	best_cf_delta	-1.672	two-sided	0.102	fdr_bh	0.008	0.61
		t-test	best_cc_delta	best_rs_delta	-3.543	two-sided	0.003	fdr_bh	0.044	29.388
		t-test	best_cf_delta	best_rs_delta	-2.209	two-sided	0.05	fdr_bh	0.017	1.494
**Figure**	**Hb**	**Test**	**F**	**p-val**	**ng2**	**eps**				
2 D	DHb	anova_rm	16.04958967	1.45E-06	0.08357575091	0.8286713646				
		**post hoc**	**A**	**B**	**T**	**Tail**	**p-val**	**corr**	**eta-square**	**BF10**
		t-test	best_cc_delta	best_cf_delta	2.145	two-sided	0.038	fdr_bh	0.015	1.329
		t-test	best_cc_delta	best_rs_delta	5.05	two-sided	0	fdr_bh	0.102	1887.497
		t-test	best_cf_delta	best_rs_delta	3.482	two-sided	0.002	fdr_bh	0.05	25.2
**Figure**	**Hb**	**Test**	**F**	**p-val**	**ng2**	**eps**				
2 E	THb	anova rm	0.690166	0.509822	0.018364	0.941664				
2 E	OHb	anova rm	0.672696	0.518386	0.010916	0.955056				
2 E	DHb	anova rm	0.14099	0.86911	0.004897	0.863214				
**Figure**	**Hb**	**cond**	**test**	**tail**	**T**	**p-val**	**power**	**BF10**		
3 A	THb	ch1	T-test	two-sided	-6.710863	2.72E-06	1	7192.454		
3 B	THb	cl1	T-test	two-sided	-10.094777	7.72E-09	1	1.58E+06		
3 C	THb	cl2	T-test	two-sided	-8.874766	5.42E-08	1	2.60E+05		

3 A	OHb	ch1	T-test	two-sided	-8.766933	6.50E-08	1	2.20E+05		
3 B	OHb	cl1	T-test	two-sided	-9.801022	1.22E-08	1	1.04E+06		
3 C	OHb	cl2	T-test	two-sided	-9.960689	9.49E-09	1	1.30E+06		
3 A	DHb	ch1	T-test	two-sided	8.570421	9.07E-08	1	1.62E+05		
3 B	DHb	cl1	T-test	two-sided	8.080983	2.12E-07	1	7.40E+04		
3 C	DHb	cl2	T-test	two-sided	5.295974	4.91E-05	1	527.727		
**Figure**	**Hb**	**Test**	**F**	**p-val**	**ng2**	**eps**				
3 D	THb	anova_rm	2.269052	0.118006	0.038296	0.6744				
3 D	OHb	anova_rm	2.266482	0.118275	0.034911	0.920718				
3 D	DHb	anova_rm	2.189169	0.126699	0.05409	0.822845				
**Figure**	**Hb**	**test**	**tail**	**T**	**p-val**	**power**	**BF10**			
3 E	THb	T-test	two-sided	-4.135410352	0.000292627372	0.9944978152	188.71			
3 E	OHb	T-test	two-sided	-2.604585851	0.01352512184	0.7389200153	4.225			
3 E	DHb	T-test	two-sided	3.536942192	0.001623564012	0.9834889618	36.582			
**Figure**	**Y**	**X**	**method**	**tail**	**r**	**p-val**	**corr**	**power**		
4 A	AQ	THb_rs	spearman	two-sided	-0.08912568744	0.6575061582	fdr_bh	0.0848300996		
4 B	AQ	THb_cf	spearman	two-sided	-0.2148616097	0.5426725338	fdr_bh	0.2684559699		
4 C	AQ	THb_cc	spearman	two-sided	-0.028328228	0.8622451739	fdr_bh	0.0530912285		
4 A	AQ	OHb_rs	spearman	two-sided	-0.1663930469	0.548662905	fdr_bh	0.1778793293		
4 B	AQ	OHb_cf	spearman	two-sided	-0.3325037526	0.2732954373	fdr_bh	0.5655757921		
4 C	AQ	OHb_cc	spearman	two-sided	-0.1295004709	0.6025285747	fdr_bh	0.1256529156		
4 A	AQ	DHb_rs	spearman	two-sided	0.1896391343	0.5426725338	fdr_bh	0.2182707866		
4 B	AQ	DHb_cf	spearman	two-sided	0.2991874977	0.2732954373	fdr_bh	0.4750679664		
4 C	AQ	DHb_cc	spearman	two-sided	0.117924484	0.6025285747	fdr_bh	0.1122415123		
**Figure**	**Y**	**X**	**method**	**tail**	**r**	**p-val**	**corr**	**power**		
4 D	AQ	THb_ch1	spearman	two-sided	-0.6687346538	0.01446356566	fdr_bh	0.8925776968		
4 E	AQ	THb_cl1	spearman	two-sided	-0.6129206333	0.0205177254	fdr_bh	0.8069164533		
4 D	AQ	OHb_ch1	spearman	two-sided	-0.2801036958	0.3903842975	fdr_bh	0.2069161503		
4 E	AQ	OHb_cl1	spearman	two-sided	-0.4031012597	0.1943584346	fdr_bh	0.3952742327		
4 D	AQ	DHb_ch1	spearman	two-sided	0.1994834439	0.4274269322	fdr_bh	0.1255757915		
4 E	AQ	DHb_cl1	spearman	two-sided	0.2387599769	0.4080103079	fdr_bh	0.1610802525		
**Figure**	**Y**	**X**	**method**	**tail**	**r**	**p-val**	**corr**	**power**		
5 A	AQ_S	THb_ch1	spearman	two-sided	-0.5917912581	0.01935319552	fdr_bh	0.7690513401		
5 B	AQ_S	THb_cl1	spearman	two-sided	-0.6566164489	0.01845705021	fdr_bh	0.8760130618		
5 A	AQ_S	OHb_ch1	spearman	two-sided	-0.3931334153	0.159807852	fdr_bh	0.3773141518		
5 B	AQ_S	OHb_cl1	spearman	two-sided	-0.5991102313	0.01935319552	fdr_bh	0.7824451917		
5 A	AQ_S	DHb_ch1	spearman	two-sided	0.1913388697	0.4469126771	fdr_bh	0.1191550365		

5 B	AQ_S	DHb_cl1	spearman	two-sided	0.3669942254	0.1609306206	fdr_bh	0.3322869059		
**Figure**	**X**	**Y**	**method**	**tail**	**r**	**p-val**	**corr**	**power**		
5 C	AQ_C	THb_ch1	spearman	two-sided	-0.6417570048	0.02454598298	fdr_bh	0.8540832004		
5 D	AQ_C	THb_cl1	spearman	two-sided	-0.3333398034	0.4627528962	fdr_bh	0.2791221623		
5 C	AQ_C	OHb_ch1	spearman	two-sided	-0.296994342	0.4627528962	fdr_bh	0.2282139392		
5 D	AQ_C	OHb_cl1	spearman	two-sided	-0.1796504237	0.7134812865	fdr_bh	0.1104870383		
5 C	AQ_C	DHb_ch1	spearman	two-sided	-0.00830753402	0.9739011846	fdr_bh	0.0493146419		
5 D	AQ_C	DHb_cl1	spearman	two-sided	-0.05192208776	0.9739011846	fdr_bh	0.0540728454		
**Figure**	**X**	**Y**	**method**	**tail**	**r**	**p-val**	**corr**	**power**		
S3	THb_rs	THb_cf	spearman	two-sided	0.4769230769	0.00239365504	fdr_bh	0.8908005329		
S3	THb_rs	THb_cc	spearman	two-sided	0.5401500938	0.00052556136	fdr_bh	0.960373298		
S3	THb_rs	OHb_rs	spearman	two-sided	0.8771106942	2.07E-12	fdr_bh	0.9999999999		
S3	THb_rs	OHb_cf	spearman	two-sided	0.6307692308	2.98E-05	fdr_bh	0.9953861393		
S3	THb_rs	OHb_cc	spearman	two-sided	0.638836773	2.54E-05	fdr_bh	0.996387892		
S3	THb_rs	DHb_rs	spearman	two-sided	-0.6399624765	2.54E-05	fdr_bh	0.996512112		
S3	THb_rs	DHb_cf	spearman	two-sided	-0.4968105066	0.00153752136	fdr_bh	0.9178746454		
S3	THb_rs	DHb_cc	spearman	two-sided	-0.4575984991	0.00358708400	fdr_bh	0.8596833853		
S3	THb_cf	THb_cc	spearman	two-sided	0.4508442777	0.00404794622	fdr_bh	0.8476971873		
S3	THb_cf	OHb_rs	spearman	two-sided	0.4686679174	0.00283870362	fdr_bh	0.878087982		
S3	THb_cf	OHb_cf	spearman	two-sided	0.8365853659	1.60E-10	fdr_bh	0.9999999762		
S3	THb_cf	OHb_cc	spearman	two-sided	0.4497185741	0.00404794622	fdr_bh	0.8456444659		
S3	THb_cf	DHb_rs	spearman	two-sided	-0.3833020638	0.01596056443	fdr_bh	0.6997108556		
S3	THb_cf	DHb_cf	spearman	two-sided	-0.1628517824	0.3153652463	fdr_bh	0.1722330029		
S3	THb_cf	DHb_cc	spearman	two-sided	-0.2583489681	0.1105587073	fdr_bh	0.3688799554		
S3	THb_cc	OHb_rs	spearman	two-sided	0.6133208255	4.92E-05	fdr_bh	0.9924295604		
S3	THb_cc	OHb_cf	spearman	two-sided	0.5362101313	0.00056666397	fdr_bh	0.9573736439		
S3	THb_cc	OHb_cc	spearman	two-sided	0.8900562852	5.60E-13	fdr_bh	1		
S3	THb_cc	DHb_rs	spearman	two-sided	-0.6131332083	4.92E-05	fdr_bh	0.9923909774		
S3	THb_cc	DHb_cf	spearman	two-sided	-0.3609756098	0.02341629746	fdr_bh	0.6421418264		
S3	THb_cc	DHb_cc	spearman	two-sided	-0.5170731707	0.00095192992	fdr_bh	0.9404305553		
S3	OHb_rs	OHb_cf	spearman	two-sided	0.6682926829	1.10E-05	fdr_bh	0.9986569814		
S3	OHb_rs	OHb_cc	spearman	two-sided	0.7857410882	1.37E-08	fdr_bh	0.9999972781		
S3	OHb_rs	DHb_rs	spearman	two-sided	-0.839587242	1.55E-10	fdr_bh	0.9999999832		
S3	OHb_rs	DHb_cf	spearman	two-sided	-0.6478424015	2.03E-05	fdr_bh	0.9972862017		
S3	OHb_rs	DHb_cc	spearman	two-sided	-0.6628517824	1.14E-05	fdr_bh	0.9983679947		
S3	OHb_cf	OHb_cc	spearman	two-sided	0.6281425891	3.03E-05	fdr_bh	0.9950145014		
S3	OHb_cf	DHb_rs	spearman	two-sided	-0.6315196998	2.98E-05	fdr_bh	0.9954880318		
S3	OHb_cf	DHb_cf	spearman	two-sided	-0.5084427767	0.00116466895	fdr_bh	0.9314267042		
S3	OHb_cf	DHb_cc	spearman	two-sided	-0.492120075	0.00167717402	fdr_bh	0.9119381243		
S3	OHb_cc	DHb_rs	spearman	two-sided	-0.7459662289	1.70E-07	fdr_bh	0.9999621175		

S3	OHb_cc	DHb_cf	spearman	two-sided	-0.5968105066	8.24E-05	fdr_bh	0.9883735858		
S3	OHb_cc	DHb_cc	spearman	two-sided	-0.6639774859	1.14E-05	fdr_bh	0.9984317428		
S3	DHb_rs	DHb_cf	spearman	two-sided	0.6112570356	5.03E-05	fdr_bh	0.9919963339		
S3	DHb_rs	DHb_cc	spearman	two-sided	0.7606003752	7.40E-08	fdr_bh	0.9999843912		
S3	DHb_cf	DHb_cc	spearman	two-sided	0.6300187617	2.98E-05	fdr_bh	0.9952823618		
**Figure**	**Hb**	**Test**	**F**	**p-val**	**np2**					
S4 A	THb	anova	1.468977	0.239274	0.109064					
S4 A	OHb	anova	1.699261	0.184489	0.124040					
S4 A	DHb	anova	0.822607	0.490014	0.064153					
**Figure**	**Hb**	**cond**	**test**	**tail**	**T**	**p-val**	**power**	**BF10**		
S4 B	THb	cl	T-test	two-sided	-7.477467661	2.98E-06	1	6535.951		
S4 C	THb	cm	T-test	two-sided	-10.78919537	3.62E-08	1	3.57E+05		
S4 D	THb	ch	T-test	two-sided	-7.367574418	3.52E-06	1	5620.865		
S4 B	OHb	cl	T-test	two-sided	-6.925854779	7.03E-06	1	3026.086		
S4 C	OHb	cm	T-test	two-sided	-8.751777275	4.74E-07	1	3.43E+04		
S4 D	OHb	ch	T-test	two-sided	-6.720772115	9.77E-06	1	2253.917		
S4 B	DHb	cl	T-test	two-sided	5.605146471	6.49E-05	1	419.525		
S4 C	DHb	cm	T-test	two-sided	8.7177353	4.97E-07	1	3.29E+04		
S4 D	DHb	ch	T-test	two-sided	7.695844802	2.14E-06	1	8787.149		
**Figure**	**Hb**	**Test**	**F**	**p-val**	**ng2**	**eps**				
S5 A	THb	anova_rm	0.142388221	0.836101161	0.002129265492	0.8574026346				
S5 A	OHb	anova_rm	0.8575287216	0.4084718668	0.012056387	0.8127922358				
S5 A	DHb	anova_rm	0.0492147813	0.9520061779	0.000880056189	0.9249617483				
**Figure**	**Hb**	**Test**	**F**	**p-val**	**ng2**	**eps**				
S5 B	THb	anova_rm	0.7901383826	0.4614933828	0.02425291708	0.9986208008				
S5 B	OHb	anova_rm	1.674003787	0.2017602374	0.04856769248	0.7542993501				
S5 B	DHb	anova_rm	3.968225339	0.02770738552	0.1392649898	0.9787464826				
		**post hoc**	**A**	**B**	**T**	**Tail**	**p-val**	**corr**	**eta-square**	**BF10**
		t-test	DHb_h1	DHb_l1	-1.730589598	two-sided	0.1509476579	fdr_bh	0.11068091	0.831
		t-test	DHb_h1	DHb_l2	-2.998867973	two-sided	0.0231130617	fdr_bh	0.16030517	6.467
		t-test	DHb_l1	DHb_l2	-0.9390763658	two-sided	0.3601231344	fdr_bh	0.02444843	0.35
**Figure**	**Hb**	**Test**	**tail**	**T**	**p-val**	**power**	**BF10**			
S5 C	THb	T-test	two-sided	0.3508377253	0.7278873157	0.0642876307	0.294			
S5 C	OHb	T-test	two-sided	2.001927035	0.05185902467	0.4570879881	1.418			
S5 C	DHb	T-test	two-sided	-0.2732887115	0.7860643734	0.05767835824	0.288			

Figure	X	Y	method	tail	r	p-val	corr	power		
S6	THb_ch1	THb_cl1	spearman	two-sided	0.6087719298	0.02268653139	fdr_bh	0.8235262665		
S6	THb_ch1	THb_cl2	spearman	two-sided	0.1631578947	0.5341931162	fdr_bh	0.1026338979		
S6	THb_ch1	OHb_ch1	spearman	two-sided	0.7964912281	0.00161675269	fdr_bh	0.9933871752		
S6	THb_ch1	OHb_cl1	spearman	two-sided	0.6561403509	0.0164324785	fdr_bh	0.8944050985		
S6	THb_ch1	OHb_cl2	spearman	two-sided	0.5245614035	0.05431644325	fdr_bh	0.6635326708		
S6	THb_ch1	DHb_ch1	spearman	two-sided	-0.3210526316	0.2162026265	fdr_bh	0.2747904283		
S6	THb_ch1	DHb_cl1	spearman	two-sided	-0.2666666667	0.3132791593	fdr_bh	0.2003626834		
S6	THb_ch1	DHb_cl2	spearman	two-sided	-0.5157894737	0.05710034105	fdr_bh	0.6454438047		
S6	THb_cl1	THb_cl2	spearman	two-sided	0.5421052632	0.04568380188	fdr_bh	0.6992161172		
S6	THb_cl1	OHb_ch1	spearman	two-sided	0.4912280702	0.06923077716	fdr_bh	0.5943320883		
S6	THb_cl1	OHb_cl1	spearman	two-sided	0.6298245614	0.01978168859	fdr_bh	0.8572071211		
S6	THb_cl1	OHb_cl2	spearman	two-sided	0.4350877193	0.1186849978	fdr_bh	0.4786541665		
S6	THb_cl1	DHb_ch1	spearman	two-sided	-0.4368421053	0.1186849978	fdr_bh	0.482182339		
S6	THb_cl1	DHb_cl1	spearman	two-sided	-0.4105263158	0.1322708867	fdr_bh	0.4302182026		
S6	THb_cl1	DHb_cl2	spearman	two-sided	-0.6754385965	0.0164324785	fdr_bh	0.9179073721		
S6	THb_cl2	OHb_ch1	spearman	two-sided	0.3385964912	0.2025171966	fdr_bh	0.3022070716		
S6	THb_cl2	OHb_cl1	spearman	two-sided	0.2	0.4491065601	fdr_bh	0.1310642523		
S6	THb_cl2	OHb_cl2	spearman	two-sided	0.6228070175	0.01978168859	fdr_bh	0.8463458018		
S6	THb_cl2	DHb_ch1	spearman	two-sided	-0.2070175439	0.4445056859	fdr_bh	0.137252612		
S6	THb_cl2	DHb_cl1	spearman	two-sided	-0.4105263158	0.1322708867	fdr_bh	0.4302182026		
S6	THb_cl2	DHb_cl2	spearman	two-sided	-0.4	0.1404293427	fdr_bh	0.4100889069		
S6	OHb_ch1	OHb_cl1	spearman	two-sided	0.5964912281	0.02367902992	fdr_bh	0.8024438713		
S6	OHb_ch1	OHb_cl2	spearman	two-sided	0.6684210526	0.0164324785	fdr_bh	0.9097432922		
S6	OHb_ch1	DHb_ch1	spearman	two-sided	-0.3824561404	0.1591267971	fdr_bh	0.3775155999		
S6	OHb_ch1	DHb_cl1	spearman	two-sided	-0.0649122807	0.8143901624	fdr_bh	0.0573681965		
S6	OHb_ch1	DHb_cl2	spearman	two-sided	-0.3701754386	0.1710024913	fdr_bh	0.355505772		
S6	OHb_cl1	OHb_cl2	spearman	two-sided	0.6403508772	0.01885484403	fdr_bh	0.8727727832		
S6	OHb_cl1	DHb_ch1	spearman	two-sided	-0.6631578947	0.0164324785	fdr_bh	0.9033323178		
S6	OHb_cl1	DHb_cl1	spearman	two-sided	-0.3333333333	0.2025171966	fdr_bh	0.2938124504		
S6	OHb_cl1	DHb_cl2	spearman	two-sided	-0.5947368421	0.02367902992	fdr_bh	0.7993529497		
S6	OHb_cl2	DHb_ch1	spearman	two-sided	-0.4192982456	0.1322708867	fdr_bh	0.447296266		
S6	OHb_cl2	DHb_cl1	spearman	two-sided	-0.350877193	0.1949191697	fdr_bh	0.3223475095		
S6	OHb_cl2	DHb_cl2	spearman	two-sided	-0.5105263158	0.05741120139	fdr_bh	0.634534799		
S6	DHb_ch1	DHb_cl1	spearman	two-sided	0.01052631579	0.9658856102	fdr_bh	0.0494386531		
S6	DHb_ch1	DHb_cl2	spearman	two-sided	0.3333333333	0.2025171966	fdr_bh	0.2938124504		
S6	DHb_cl1	DHb_cl2	spearman	two-sided	0.5736842105	0.03066774314	fdr_bh	0.7608717253		
**Figure**	**Y**	**X**	**method**	**tail**	**r**	**p-val**	**corr**	**power**		
S7 A	AQ_A	THb_ch1	spearman	two-sided	-0.2281350257	0.77369489	fdr_bh	0.1507192207		
S7 B	AQ_A	THb_cl1	spearman	two-sided	-0.1010461073	0.77369489	fdr_bh	0.0679236948		
S7 A	AQ_A	OHb_ch1	spearman	two-sided	-0.07291987122	0.77369489	fdr_bh	0.0588648849		

S7 B	AQ_A	OHb_cl1	spearman	two-sided	-0.1375060429	0.77369489	fdr_bh	0.0843789224		
S7 A	AQ_A	DHb_ch1	spearman	two-sided	0.1958419399	0.77369489	fdr_bh	0.1226659826		
S7 B	AQ_A	DHb_cl1	spearman	two-sided	-0.2375104377	0.77369489	fdr_bh	0.1598321171		
**Figure**	**Y**	**X**	**method**	**tail**	**r**	**p-val**	**corr**	**power**		
S7 C	AQ_I	THb_ch1	spearman	two-sided	-0.3709176048	0.3925638006	fdr_bh	0.3388451079		
S7 D	AQ_I	THb_cl1	spearman	two-sided	-0.369869815	0.3925638006	fdr_bh	0.3370865347		
S7 C	AQ_I	OHb_ch1	spearman	two-sided	0.03352927501	0.8949238024	fdr_bh	0.0512206411		
S7 D	AQ_I	OHb_cl1	spearman	two-sided	-0.2776643087	0.3969072558	fdr_bh	0.2039643689		
S7 C	AQ_I	DHb_ch1	spearman	two-sided	0.2179402876	0.4619653399	fdr_bh	0.1413099487		
S7 D	AQ_I	DHb_cl1	spearman	two-sided	0.2975723157	0.3969072558	fdr_bh	0.2289693136		
**Figure**	**Y**	**X**	**method**	**tail**	**r**	**p-val**	**corr**	**power**		
S7 E	AQ_D	THb_ch1	spearman	two-sided	-0.3272801904	0.5548063197	fdr_bh	0.2701568168		
S7 F	AQ_D	THb_cl1	spearman	two-sided	-0.4561142971	0.3426507414	fdr_bh	0.49687182		
S7 E	AQ_D	OHb_ch1	spearman	two-sided	-0.1516917708	0.657552151	fdr_bh	0.0922901976		
S7 F	AQ_D	OHb_cl1	spearman	two-sided	-0.112210351	0.657552151	fdr_bh	0.0723840379		
S7 E	AQ_D	DHb_ch1	spearman	two-sided	0.1184442594	0.657552151	fdr_bh	0.0750940848		
S7 F	AQ_D	DHb_cl1	spearman	two-sided	0.1911731906	0.657552151	fdr_bh	0.1190276925		

Supplementary Table 2 (Table S2). Descriptive statistic of AQ total score and subscales (AQ_S, AQ_C, AQ_A, AQ_D, AQ_I) for adult population.

Adults	Mean	Standard Deviation	Min	25%	50%	75%	Max	Kurtosis	Skewness	Shapiro-Wilk Test	p-value
AQ	15.075	6.553087	3	11	14	19	32	0.994083	0.885477	0.937869	0.029325
AQ_S	2.025	2.325857989	0	0	1	3	10	2.257498496	1.499853609	0.810435653	1.13E-05
AQ_C	1.9	1.706699	0	1	2	2.25	8	3.469255	1.596856	0.843179	6.17E-05
AQ_A	4.625	1.764137038	1	4	5	6	9	0.281890889	-0.070514356	0.951339304	0.084325403
AQ_D	4.45	2.611120204	0	2	4	6.25	10	-0.778814946	0.20526636	0.965760052	0.262098789
AQ_I	2.075	1.831175887	0	1	2	3	8	2.069518058	1.413454839	0.848571181	8.31E-05

Supplementary Table 3 (Table S3). Descriptive statistic of AQ total score and subscales (AQ_S, AQ_C, AQ_A, AQ_D, AQ_I) for child population.

Children	Mean	Standard Deviation	Min	25%	50%	75%	Max	Kurtosis	Skewness	Shapiro-Wilk Test	p-value
AQ	32.11111	10.66544	17	24.25	30	41.5	49	-1.4335	0.172442	0.926583	0.169045
AQ_S	4.888888889	3.676457516	1	2	4.5	7.5	12	-0.944211415	0.570800869	0.886443734	0.033554446
AQ_C	5.722222222	3.044871398	1	3.25	5	8	11	-0.959912079	0.36740446	0.942008555	0.313533425
AQ_A	6.333333333	3.124852938	1	4	6	9.5	11	-1.197267383	0.133018308	0.929846883	0.192989156
AQ_D	9.333333333	4.352146191	0	6.25	9.5	12.75	19	0.720012731	-0.00856292	0.972668886	0.846108854
AQ_I	5.833333333	2.455486055	3	4	6	7	10	-0.794560381	0.537869765	0.889475226	0.037801977
